# *One pattern analysis* (*OPA*) for the quantitative determination of protein interactions in plant cells

**DOI:** 10.1186/s13007-023-01049-3

**Published:** 2023-07-28

**Authors:** Jan Eric Maika, Benedikt Krämer, Vivien I. Strotmann, Frank Wellmer, Stefanie Weidtkamp-Peters, Yvonne Stahl, Rüdiger Simon

**Affiliations:** 1 Institute for Developmental Genetics and Cluster of Excellence on Plant Sciences, Heinrich Heine University, Universitätsstraße 1, 40225 Düsseldorf, Germany; 2 PicoQuant GmbH, Rudower Chaussee 29 (IGZ), 12489 Berlin, Germany; 3 Smurfit Institute of Genetics, Trinity College Dublin, Dublin, Ireland; 4 Centre for Advanced Imaging, Heinrich Heine University, Universitätsstraße 1, 40225 Düsseldorf, Germany

## Abstract

**Background:**

A commonly used approach to study the interaction of two proteins of interest (POIs) in vivo is measuring Förster Resonance Energy Transfer (FRET). This requires the expression of the two POIs fused to two fluorescent proteins that function as a FRET pair. A precise way to record FRET is Fluorescence Lifetime IMaging (FLIM) which generates quantitative data that, in principle, can be used to resolve both complex structure and protein affinities. However, this potential resolution is often lost in many experimental approaches. Here we introduce a novel tool for FLIM data analysis of multiexponential decaying donor fluorophores, *one pattern analysis (OPA),* which allows to obtain information about protein affinity and complex arrangement by extracting the relative amplitude of the FRET component and the FRET transfer efficiency from other FRET parameters.

**Results:**

As a proof of concept for *OPA*, we used FLIM-FRET, or FLIM-FRET in combination with BiFC to reassess the dimerization and tetramerization properties of known interacting MADS-domain transcription factors in *Nicotiana benthamiana* leaf cells and *Arabidopsis thaliana* flowers. Using the *OPA* tool and by extracting protein BINDING efficiencies from FRET parameters to dissect MADS-domain protein interactions in vivo in transient *N. benthamiana* experiments, we could show that MADS-domain proteins display similar proximities within dimeric or tetrameric complexes but bind with variable affinities. By combining FLIM with BiFC, we were able to identify SEPALLATA3 as a mediator for tetramerization between the other MADS-domain factors. *OPA* also revealed that in vivo expression from native promoters at low levels in *Arabidopsis* flower meristems, makes in situ complex formation of MADS-domain proteins barely detectable.

**Conclusions:**

We conclude that MADS-domain protein interactions are transient in situ and may involve additional, so far unknown interaction mediators. We conclude that *OPA* can be used to separate protein binding from information about proximity and orientation of the interacting proteins in their complexes. Visualization of individual protein interactions within the underlying interaction networks in the native environment is still restrained if expression levels are low and will require continuous improvements in fluorophore labelling, instrumentation set-ups and analysis tools.

**Supplementary Information:**

The online version contains supplementary material available at 10.1186/s13007-023-01049-3.

## Introduction

**Fig. 1:**
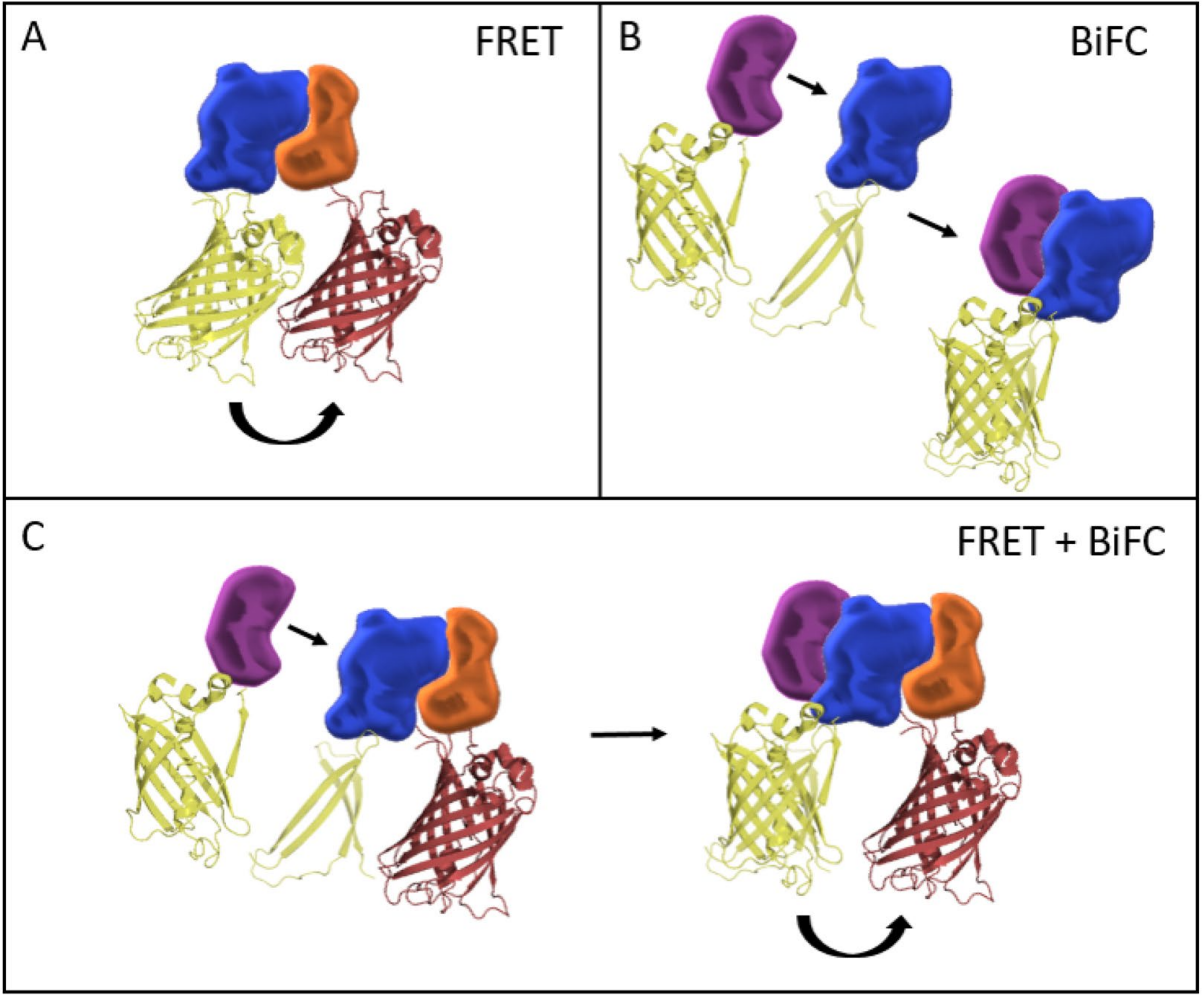
FRET and BiFC can be used to investigate protein–protein interactions. **A** FRET describes the process of non-radiative energy transfer from an excited “donor” fluorophore to a non-excited “acceptor” molecule. Occurrence of FRET depends on three specific conditions: (i) the emission spectrum of the donor and the excitation spectrum of the acceptor must sufficiently overlap. (ii) The orientation of donor and acceptor dipoles must not be oriented perpendicular to each other. (iii) Donor and acceptor molecules must be in close proximity towards each other (< 1 nm or 100 Å distance). **B** BiFC is based on the complementation of two fragments of a fluorescence protein (FP). Fluorophore functionality is regained when the fragments, fused to interacting proteins, are brought in proximity to each other. **C** Combination of BiFC with FRET allows to investigate larger protein complexes. Thereby, the complemented FP can serve as a donor or acceptor

Protein interactions and the formation of higher-order protein complexes play a crucial role in a plethora of cellular and developmental processes, but the precise identification and monitoring of protein–protein interactions (PPIs) in cells remains challenging. Two common techniques to visualize and quantify protein–protein interactions in vivo are BiFC and FRET (Fig. 1A and B).

**Fig. 2:**
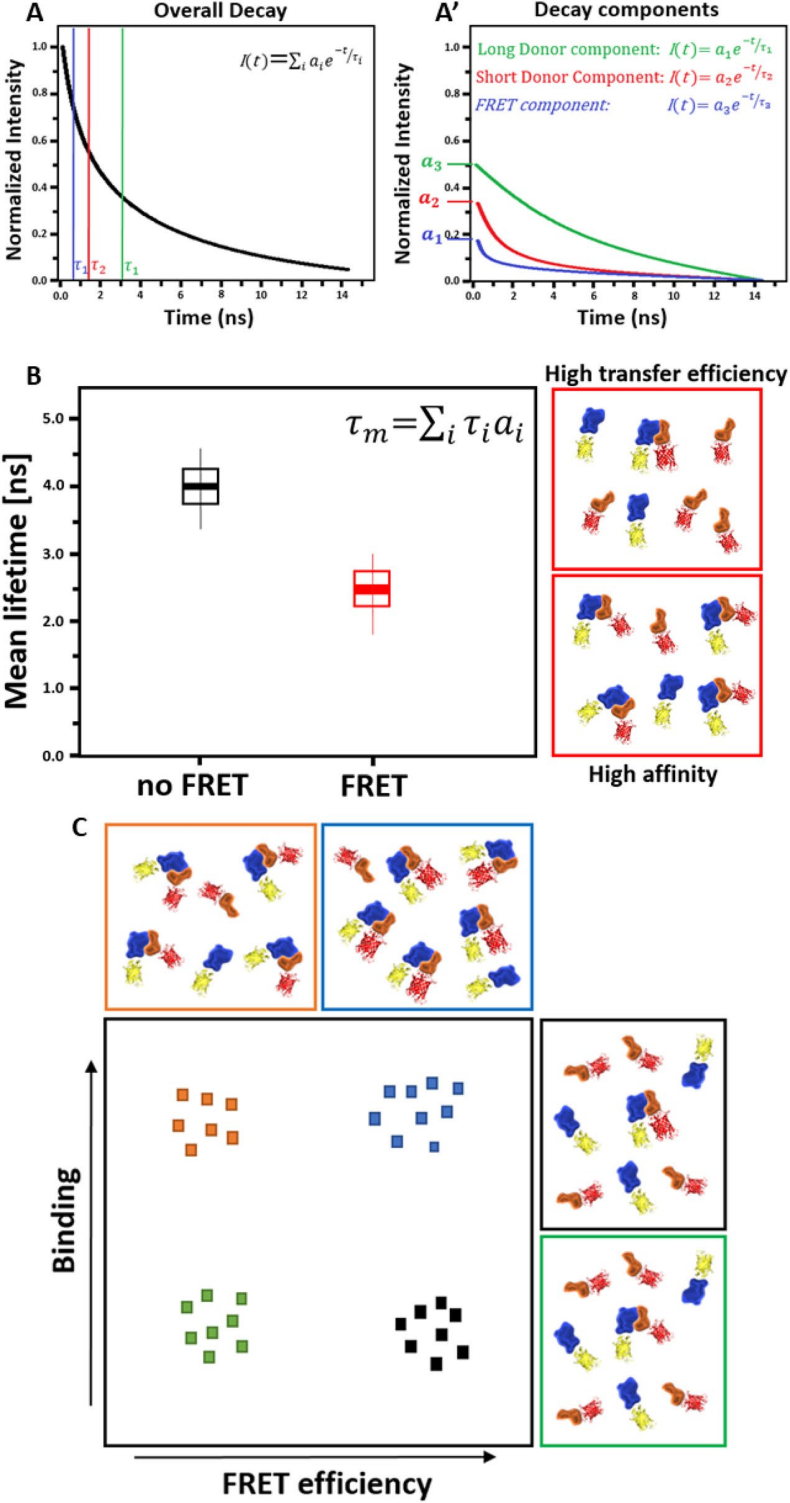
BINDING and FRET efficiency in FLIM experiments. **A** Schematic of a multiexponential fluorescence decay when FRET occurs. Fluorescence lifetime τ is defined as the average time a fluorophore stays in its excited state. During this time, the intensity *I(t)* decreases by 63.8%. The decaying intensity at time *t* is given by the summed decay functions across all components *i*, where $\tau$ is the lifetime and $\alpha$ the pre-exponential factor (amplitude) of the exponential decay function. **A**’ Schematic of the different components in the overall decay. The sum of the individual components would result in the overall decay curve. **B** The mean amplitude weighted lifetime ${\tau }_{m}$ of a mixture of differentially decaying components is given by the sum of each component’s lifetime (${\tau }_{i}$) weighted by its respective amplitude (${\alpha }_{i}$). In case of FRET, ${\tau }_{m}$ decreases and can be used as a measure for protein–protein interaction. However, reduction of ${\tau }_{m}$ ccould be due to high affinity between the proteins and low energy transfer efficiency between fluorophores or vice versa. Therefore, using ${\tau }_{m}$, one loses information which are usually included in FLIM data. **C** Using the amplitudes and the lifetimes of the exponential decay, BINDING and FRET efficiency can be calculated. BINDING is indicative of how many molecules undergo FRET in a sample, whereas FRET efficiency describes the efficiency of the energy transfer between the fluorophores. As energy transfer efficiency is dependent on the distance between fluorophores and the orientation of their dipoles, FRET efficiency can be used as a measure for proximity and orientation within the complex and BINDING as an indicator for the affinity between the proteins. Increase of BINDING correlates with more protein–protein interactions. Increase in FRET efficiency indicates higher transfer efficiency due to higher proximity of the fluorophores and similar orientation of their dipoles

BiFC is based on the complementation of two fragments of a fluorescence protein (FP; Fig. 1B). Fluorophore functionality is regained when the fragments, fused to interacting POIs, are brought in close proximity to each other [15]. FRET describes the process of non-radiative energy transfer from an excited “donor” fluorophore to an “acceptor” molecule [9]. Occurrence of FRET depends on three specific conditions: (i) The emission spectrum of the donor and the excitation spectrum of the acceptor must sufficiently overlap. (ii) The orientation of donor and acceptor dipoles must not be oriented perpendicular to each other. (iii) Donor and acceptor molecules must be in close proximity to each other (< 10 nm or 100 Å distance). FRET is considered the more accurate method and less susceptible to false positive interactions when compared to BiFC [2, 13, 41]. Commonly, FRET is measured either by fluorescence intensity-based techniques such as FRET-Acceptor Photo Bleaching (APB) and recording of sensitized emission, or by the analysis of the fluorescence lifetime of donor fluorescence using FLIM. Intensity-based FRET usually requires strict controls and correction for spectral bleed-through, whereas lifetime acquisition by FLIM is more robust [2, 11, 31, 39, 41]. Additionally, FLIM-FRET is independent from local Donor and Acceptor concentrations and requires only relatively low irradiation of cells, and is thus considered to be superior compared to intensity-based techniques [7, 10, 26, 27, 35, 40]. In time-domain FLIM experiments, arrival times of single photons after excitation with a pulsed laser are recorded and binned into a histogram, resulting in a characteristic fluorescence decay for a specific fluorophore. The fluorescence lifetime τ is the average time such fluorophore stays in its excited state, whereas during this time, its intensity decreases by ~ 64% [5]. Decaying intensity at time *t* is given by the summed decay functions across all components *i*, where $\tau$ is the lifetime and $\alpha$ the pre-exponential factor (amplitude) of the exponential decay function (Fig. 2A).

Occurrence of FRET leads to quenching of donor intensity and a decrease of its lifetime. Thus, in the overall decay of a bi-exponential donor fluorophore, a third component describes the effect of FRET. In this case, each of the three decay components have their own lifetime and amplitude. Thereby, the lifetimes ${\tau }_{i}$ of the individual components describe the decay rate, and the amplitudes ${\alpha }_{i}$ describe the contribution of each component to the overall decay (Fig. 2A’). Commonly, the average amplitude weighted lifetime ${\tau }_{m}$ of a mixture of differentially decaying components is calculated by the sum of each component’s lifetime (${\tau }_{i}$), weighted by its respective amplitude (${\alpha }_{i}$). In case of FRET, ${\tau }_{m}$ decreases and can be used as a measure for PPI (Fig. 2B).

Importantly, reduction of ${\tau }_{m}$ can be due to either (i) high affinity between the proteins but low proximity, resulting in low energy transfer efficiency between fluorophores, or (ii) low protein affinity but high proximity, resulting in high energy transfer efficiency. Thus, the same value of ${\tau }_{m}$ can either be a result of a high number of interacting proteins but with low proximity or a lower number of interacting proteins but with high proximity and thus more effective transfer of energy between fluorophores. This crucial information about binding affinities and spatial information within the complex is in principle available within the acquired FLIM data and can be accessed by analysing amplitudes and lifetimes separately. Consequently, the relative amplitude of the FRET fraction, here termed BINDING, and the FRET efficiency, based on the reduction of the FRET component lifetime compared to the donor component lifetime can be determined. Thereby, BINDING is indicative for the relative number of molecules undergoing FRET in a sample, whereas FRET efficiency describes the efficiency of the energy transfer between the fluorophores. As energy transfer efficiency is dependent on the distance between fluorophores and the orientation of their dipoles [9], FRET efficiency can be used as a measure for proximity and orientation within the complex [4] and BINDING as an indicator for the affinity between the proteins (Fig. 2C). While calculation of BINDING (relative amplitude of the FRET fraction) is trivial for monoexponentially decaying donors and was described before [30, 50], determination of BINDING parameter from decays of multiexponential decaying donors is more difficult. As the fluorescent protein mVenus can display a biexponentially decaying behaviour [36], we here apply a newly developed analysis method, “*One Pattern Analysis* (*OPA*)” (PicoQuant, Berlin, Parts of this method is covered by a German patent application DE10 2021 107 759.1), in which the donor only decay components (Donor only lifetime components and their respective amplitudes) are pre-defined as a “pattern”, allowing the calculation of BINDING from multiexponential donor decays. By discriminating between the affinity of interacting proteins and their proximity or orientation within the forming protein complex, we reassessed dimer and tetramer formation of MADS-domain transcription factors involved in the specification of floral organs *in planta*. The activities of these floral regulators is summarized in the Floral Quartet Model (FQM) [44], which posits that tetrameric complexes of MADS-domain proteins bind to proximal CArG-box sequences (CArG: C-A-rich-G; consensus: 5ʹ-CC(A/T)_6_GG-3ʹ) to regulate their target genes.

Thus, for the specific development of each floral organ, different tetrameric complexes of MADS-domain proteins are responsible [12, 43, 44]. However, while tetramerization seems to be characteristic for MADS-domain proteins, chromatin immunoprecipitation experiments followed by next generation sequencing also highlighted the relevance of dimers and it is assumed that both, tetramers and dimers, can occur in dynamic equilibria [21, 22, 22, 23, 23].

Over the past three decades, complex formation of plant MADS-domain proteins has been well characterised and numerous complex combinations have been reported [8, 12, 14, 16–18, 20, 28, 34, 38, 47, 49]. (A summary of Arabidopsis MADS-domain protein interactions can be found in Table 1)Thus, the FQM is well supported, but yet little is known about the stoichiometry or the presence of distinct complexes *in planta*. Although previously reported FRET assays in *N. benthamiana* leaf cells and *Arabidopsis* protoplasts support the idea of in vivo complex formation [14, 17, 18, 28], they come with the limitation that they do not resolve binding dynamics or PPI strength. Additionally, because standard two-colour FRET and BiFC are limited to investigate dimeric interactions, evidence for tetramer formation *in planta* is still sparse. Initial in situ interactions have been illustrated with BiFC experiments in developing flowers [38], but equilibria between dimers and tetramers in a cellular, tissue-specific or developmental/temporal context are still not understood. Furthermore, BiFC itself affects the nature of protein interactions so that even very transient or by-chance encounters of overexpressed proteins are stabilised, creating a background of protein interactions that are unlikely to represent the in vivo situation.

**Table 1:** Overview of observed interactions between the MADS-domain proteins AP1, SEP3, PI and AP3. (Yeast 2 Hybrid (Y2H), Electrophoretic Mobility Shift Assay (EMSA), Immunoprecipitation (IP), Liquid chromatography–mass spectrometry (LC–MS)).

	Y2H	EMSA or IP	BiFC	LC–MS	FRET	Crystal structure	Literature
AP3-PI-SEP3-AG	●	●					[12, 16]
AP3-PI-SEP3-AP1					●		This study
AP3-PI-AP1	●	●			●		[12], This study
AP3-PI-SEP3	●	●			●		[12, 18], This study
PI-PI		●			●		[34], This study
AP3-PI		●	●	●	●		[34, 38, 47], This study
AP3-AP3		●			●		[34], This study
SEP3-AP3		●		●	●		[18, 38], This study
SEP3-PI		●		●	●		[18, 38], This study
SEP3-SEP3		●			●	●	[16, 18, 33], This study
AP1-AP3		●		●	●		[34, 38], This study
AP1-PI		●		●	●		[34, 38], This study
AP1-SEP3	●	●	●		●		[12, 18, 38], This study
AP1-AP1		●			●		[34], This study

To overcome these limitations, we established a pipeline using FRET-FLIM alone or in combination with BiFC (Fig. 1C) as well as discrimination between BINDING and FRET efficiency to elucidate dimer and tetramer formation between MADS-domain transcription factors more precisely. We observed strong interactions between the MADS-domain proteins APETALA1 (AP1), SEP3, PISTILLATA (PI) and APETALA3 (AP3), and the formation of tetrameric complexes in vivo using an inducible heterologous expression system (*N. benthamiana*). We were able to dissect preferences for homo- or heteromer formation between the individual complex components and found that the interaction of AP1 with AP3 and PI in the tetrameric assembly depends on SEP3. The *OPA* approach allowed us to overcome biexponential donor-only decays, which poses a general difficulty in FLIM data fitting procedures and often results in data over interpretation or erroneous determination of FRET. We noted that currently available FLIM setups do not allow to reliably detect the aforementioned tetrameric complexes in young *Arabidopsis* flowers, where the presence of other native interaction partners, variable donor or acceptor concentrations and low photon numbers lead to a diluted FRET component. Nevertheless, the development of new labelling technologies and advancement of brighter fluorophores will allow the successful determination of multimeric protein interaction networks in vivo in the future.

## Results

### Observation of AP1 and SEP3 homo- and heteromeric complexes in vivo

**Fig. 3:**
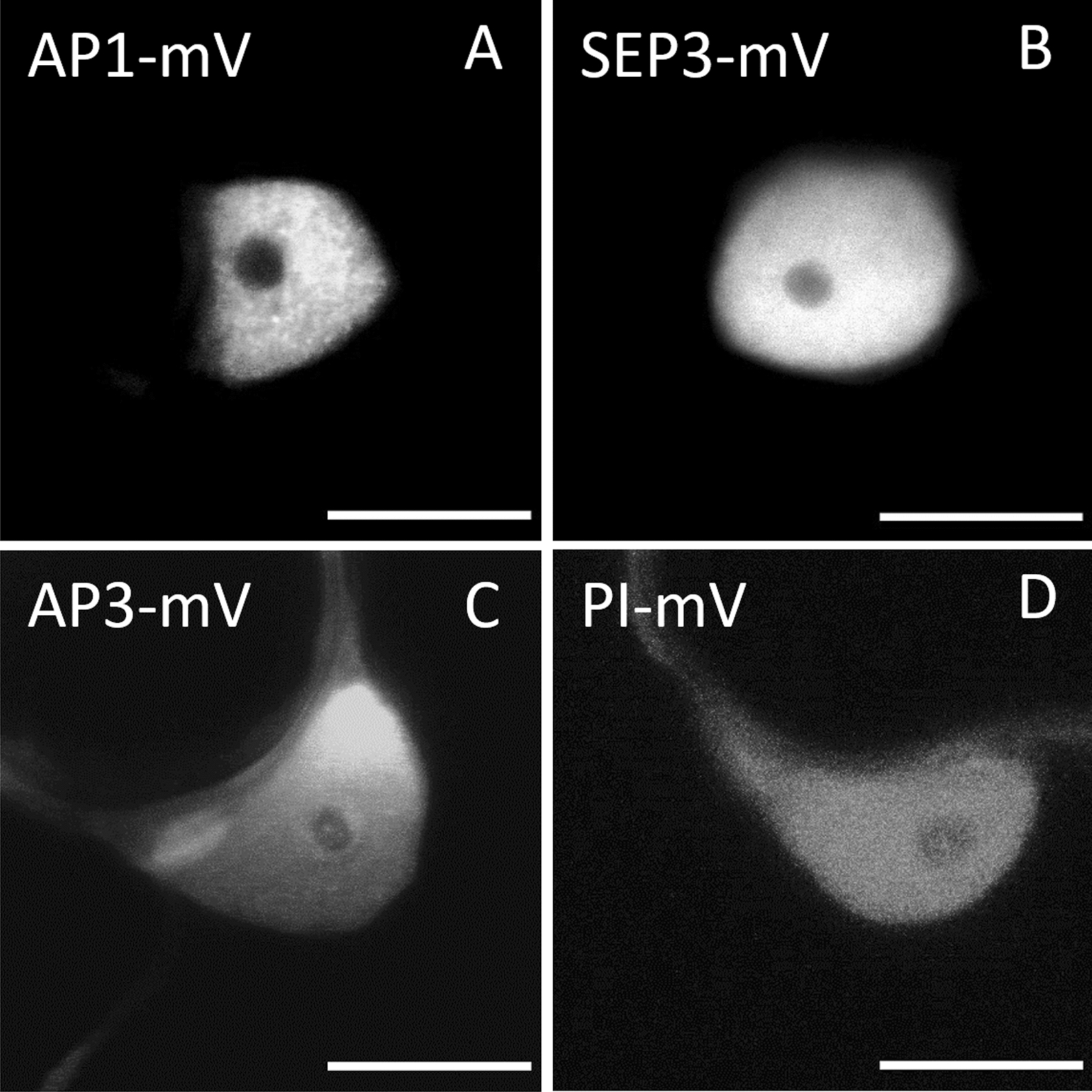
Localisation of MADS-domain proteins in *N. benthamiana* leaf cells. MADS-domain proteins fused to fluorescent proteins were transiently expressed via the UBQ10 promoter in epidermis cells of *N. benthamiana* leaves. **A** Localization of AP1-mV. **B** Localisation of SEP3-mV. AP1 and SEP3 are localised to the nucleus. **C** Localisation of AP3-mV. **D** Localisation of PI-mV. Signal from AP3 and PI was selected in both nucleus and cytoplasm. All proteins were absent from the nucleolus (Scale bars: 10 µm)

To characterize the interaction properties of MADS-domain transcription factors in vivo, we expressed C-terminal fusions between AP1, SEP3, AP3 or PI and the fluorescent proteins (FP), mVenus (mV) and mCherry (mCh), respectively, in epidermal leaf cells of transiently expressing *N. benthamiana*. When expressed individually, all fusion proteins localized to the nucleoplasm and were excluded from the nucleolus (Fig. 3A–D).

Signal of AP3-mV and PI-mV was also detected in the cytoplasm (Fig. 3C, D), as dimerization of AP3 with PI is necessary for complete nuclear localisation [17, 29]. We then measured FRET to analyse distinct complexes at a subcellular level. To this end, we acquired FLIM images of nuclei from cells expressing different combinations of the four aforementioned fusion proteins. In most cases, the measured mVenus (donor only) data displayed a bi-exponential decay consisting of a longer lifetime of ~ 3 ns and a shorter lifetime of ~ 1–2 ns. This bi-exponential decay behavior was previously described for mVenus, but also for other fluorescence proteins like YFP or GFP [1, 36, 42].Even though the amplitude of the shorter lifetime fraction is much lower compared to the amplitude of the longer lifetime fraction, we considered this as a real contribution to the decay and accordingly applied a newly developed fitting routine for multi-exponential decays to avoid artificially increased BINDING or FRET efficiencies. We applied the same fitting procedure, used for FRET samples containing both mV and mCh, to all donor only samples expressing only mVenus fused to one of the MADS-box proteins to characterize the background BINDING levels we could expect from our fitting model.

**Fig. 4:**
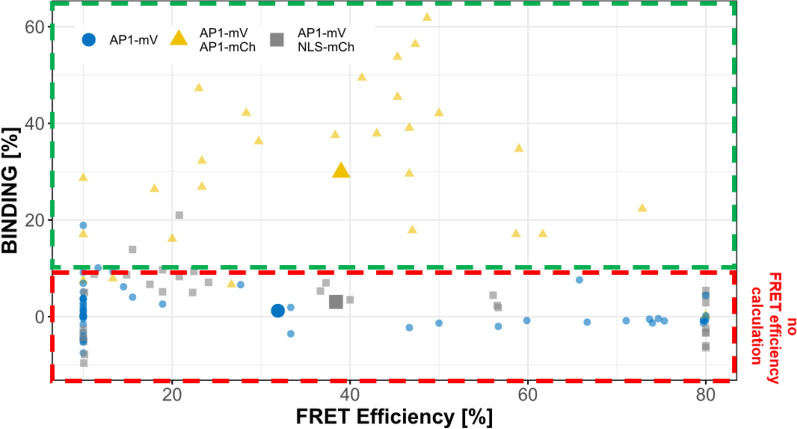
BINDING versus FRET efficiencies in FRET and no-FRET samples. BINDING and FRET efficiencies for donor only (AP1-mV), negative control (AP1-mV NLS-mCh) and FRET (AP1-mV AP1-mCh) samples. In samples where molecules don’t undergo FRET, acquired BINDING usually was between − 10 and 10%. We therefore defined this range below 10% as “limit for FRET efficiency calculation”. When BINDING was below 10%, FRET efficiencies were highly variable and accumulated at the limits (10% and 80% FRET efficiency) which defined in the fitting model. Therefore, we excluded FRET efficiencies when BINDING was below 10% and only include them when molecules in a sample undergo FRET (BINDING above 10%)

In most of the cells of the donor only sample AP1-mV we acquired apparent BINDING values between − 10 and 10% (1.2% ± 4.5; Additional file 1: Fig. S1; Fig. 4).

**Fig. 5:**
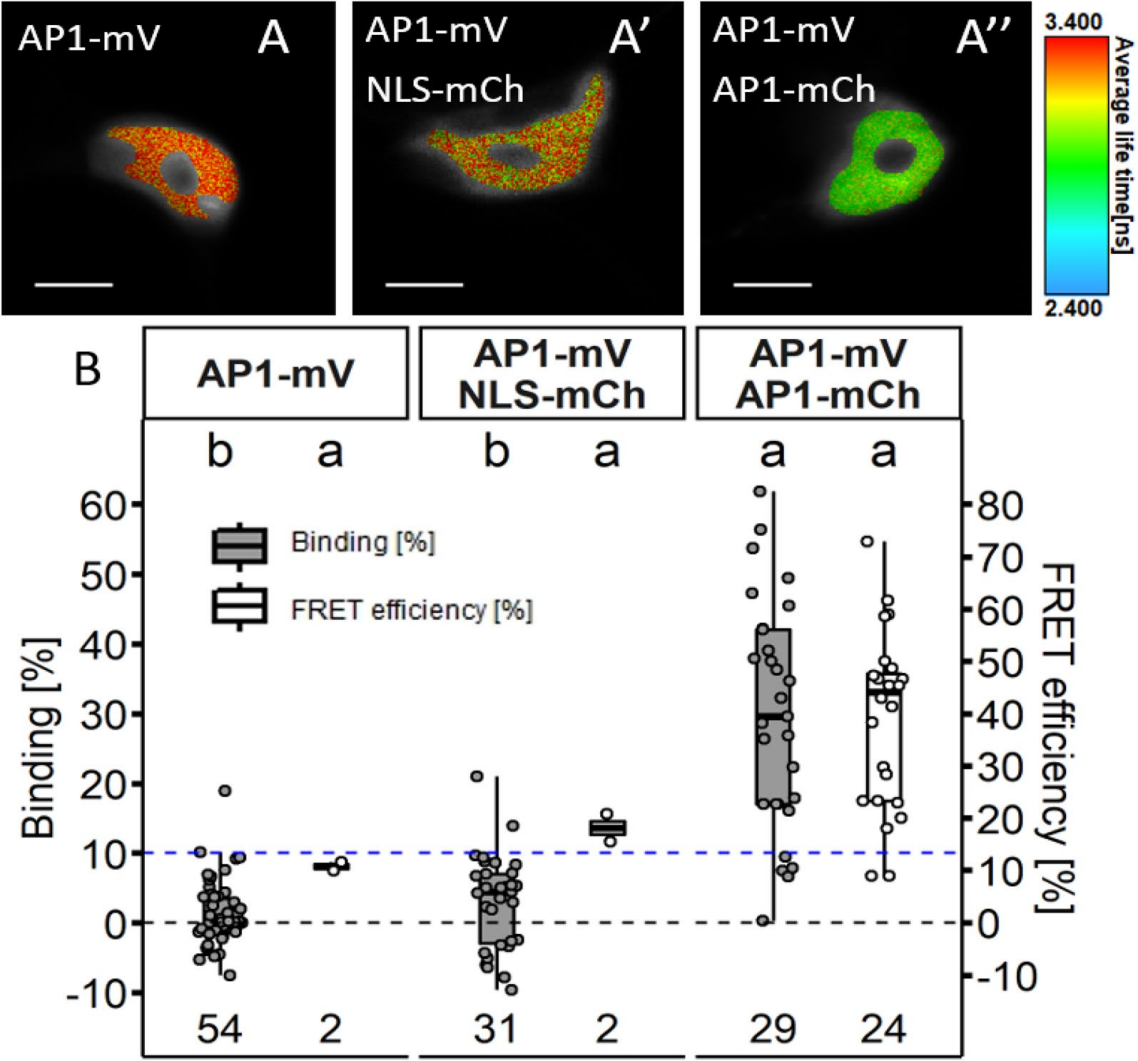
FLIM analysis of AP1-mV NLS-mCh and AP1-mV Ap1-mCh in *N. benthamiana* leaf cells. FLIM experiments were performed in *N. benthamiana* leaf epidermis cells. Fusion proteins were expressed from the *UBQ10* promoter and imaged 3–4 days after infiltration. **A** Average lifetime image of individual nuclei expressing AP1-mV, AP1-mV NLS-mCh and Ap1-mV AP1-mCh (**A**’–**A**’’ respectively). Nucleoli were excluded from FLIM analysis. (Scale bars: **A**–**A**’’: 6 µm) **B** BINDING [%] (grey) and FRET efficiencies [%] (white) for AP1-mV, AP1-mV NLS-mCh and AP1-mV AP1-mCh. For each analysed nucleus average BINDING and a corresponding average lifetime were fitted. Mean BINDING of the donor only sample AP1-mV was 1.23% and most values were below 10%. Therefore, nuclei with BINDING below 10% were excluded from FRET efficiency calculation. Co-expression of NLS-mCh with AP1-mV did not lead to significant higher BINDING (3.06%) compared to the donor only sample, while AP1-mV AP1-mCh showed increased BINDING (29.89%) with an average FRET efficiency of 39.98%. Statistical groups were assigned after multiple comparison with Kruskal–Wallis and a Post hoc test using the criterium Fisher’s least significant difference (alpha parameter is 0.05). (Dashed blue line marks the BINDING cut-off of 10%; Number of repetitions are indicated below BINDING values and number of images with BINDING above 10% are indicated below the FRET efficiency values in the bottom of the plot)

When donor only samples AP1-mV were fitted using a model, which assumes a mono exponentially decaying donor, BINDING appeared significantly increased and in one fourth of the images BINDING was above 10% (7.6% ± 9.0; Additional file 1: Fig. S2). Donor only decays must therefore be carefully examined to avoid that a second, more rapidly decaying donor component is considered as FRET. The corresponding apparent FRET efficiencies displayed high variance (Additional file 1: Table S1), usually values close to the minimum or maximum limits of our fitting model (Additional file 1: Fig. S1; Fig. 4). As in donor only samples no photons are detected originating from the FRET process, their contribution to the overall decay cannot be fitted correctly due to low photon statistic. Therefore, we acknowledged that we cannot sufficiently calculate reliable apparent FRET efficiencies for nuclei with BINDING below 10%. Consequently, we defined a cut-off for BINDING below 10% as “limit for FRET efficiency calculation”. As in this range, FRET efficiencies cannot be calculated adequately, we subsequently did not display FRET efficiencies for nuclei with lower BINDING (Fig. 5B). In nuclei displaying BINDING above the “limit for FRET efficiency calculation” of 10%, we could more reliably fit FRET efficiency and therefore subsequently display FRET efficiencies determined from these samples (Figs. 4, 5B). Indeed, when AP1-mCh was co-expressed as acceptor for AP1-mV, we measured significantly increased BINDING (29.9% ± 16.4; Fig. 5B), showing the formation of AP1 homomeric complexes (with a FRET efficiency of 39.0% ± 16.9).

Subsequent bleaching of the acceptor molecules led to a significantly decreased BINDING in the same nuclei (from 19.2% ± 4.7 to 1.17% ± 1.2; Additional file 1: Fig. S3), confirming FRET between AP1-mV and AP1-mCh. As expected, BINDING weakly correlated with the acceptor concentration (Additional file 1: Fig. S4). FRET can also occur between non-interacting proteins. This phenomenon is known as bystander FRET and is due to high protein concentrations in the analysed environment [3, 24]. To analyse a possible effect of bystander FRET in our set-up we co-expressed AP1-mV with mCh tagged to a nuclear localisation sequence (NLS). We observed a small, but not significant increase in BINDING compared to the donor only sample (from 1.2% ± 4.5 to 3.1% ± 6.8). As BINDING was not significantly elevated and in most AP1-mV NLS-mCh images below 10%, we assumed that bystander FRET could be neglected in our experimental set-up (Additional file 1: Fig. S2).

**Fig. 6:**
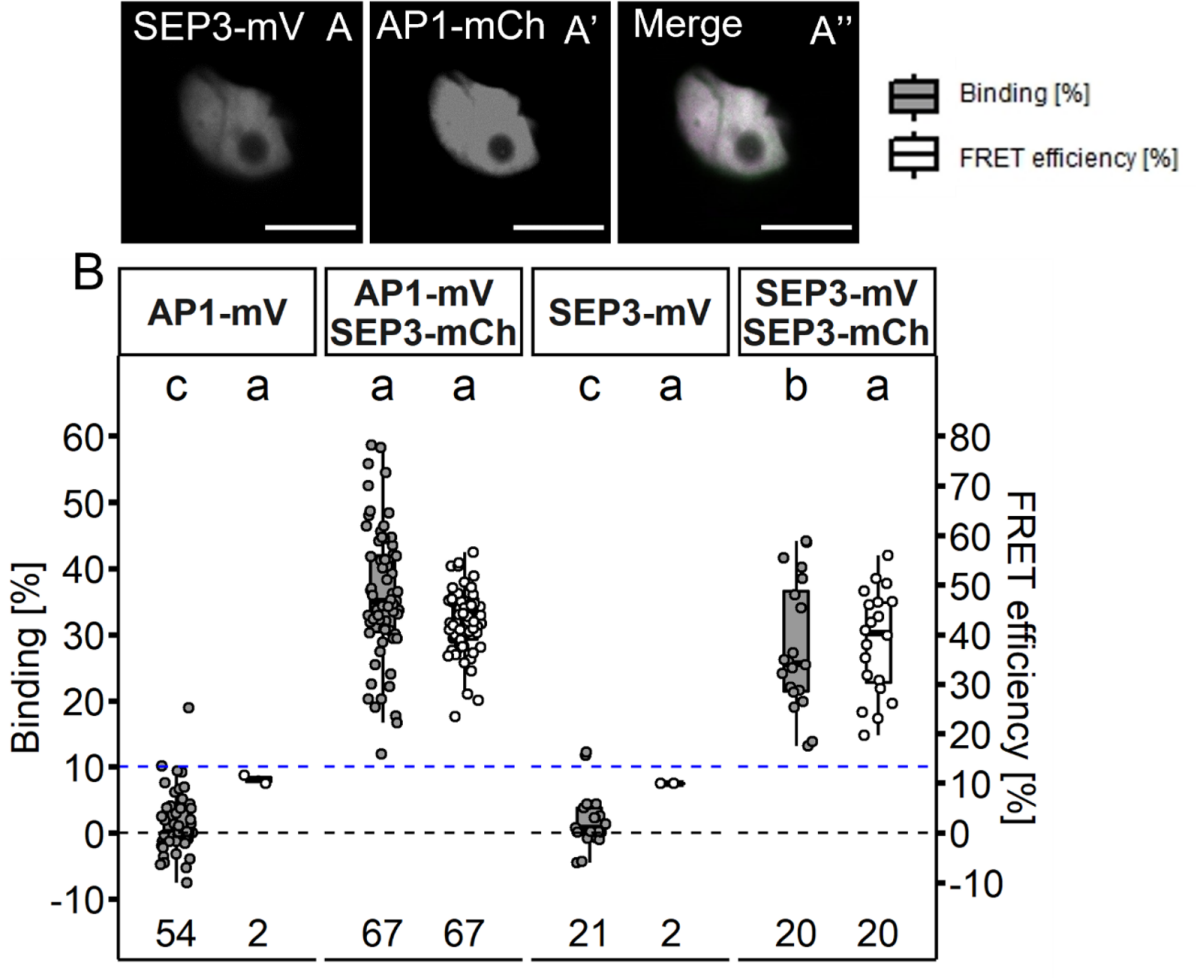
Interaction between AP1 and SEP3 proteins in *N. benthamiana* leaf cells. **A**–**A**’’ Co-localisation of SEP3-mV an AP1-mCh in *N. benthamiana* leaf cells (**A** SEP3-mV signal. **A**’ AP1-mV signal. **A**’’ Merged signal). Co-expression did not lead to a change of localisation. (Scalebars: **A**–**A**’’: 10 µm) **B** BINDING [%] (grey) and FRET efficiencies [%] (white) for AP1-mV, AP1-mV SEP3-mCh, SEP3-mV and SEP3-mV SEP3-mCh. Analysis was done as described in Fig. 5. Mean BINDING (28.15% ± 9.7) and FRET Efficiency (38.53% ± 10.53) measured for SEP3 homomers were comparable to the values obtained for AP1 homomers (compare to Fig. 5). Mean BINDING of the AP1/SEP3 heteromer (36.06% ± 10.01) was slightly increased compared to both individual homomers. Mean FRET Efficiency of the heteromers (42.64% ± 6.26) was not significantly different compared to the SEP3 homomers. Statistical groups were assigned after multiple comparison with Kruskal–Wallis and a Post hoc test using the criterium Fisher’s least significant difference (alpha parameter is 0.05) (Dashed blue line marks the BINDING cut-off of 10%; Number of repetitions are indicated below BINDING values and number of images with BINDING above 10% are indicated below the FRET efficiency values in the bottom of the plot)

According to the FQM, a quaternary complex, consisting of a dimer formed by AP1 and SEP3 that interacts with a dimer formed by AP3 and PI is responsible for the specification of petals during floral development [12, 44]. Before analysing tetramer formation between AP1, AP3, PI and SEP3 we wanted to test the stability of the individual proposed dimeric interactions. To this end, we first assessed AP1/SEP3 heteromers. Subcellular localisation of AP1 and SEP3 did not change upon co-expression (Fig. 6A–A’’).

BINDING values of AP/SEP3 (36.1% ± 10.0; Fig. 6B) showed high affinity between the two proteins, suggesting stable dimer or even tetramer formation. Mean FRET efficiency of AP1/SEP3 (42.6% ± 6.3) was comparable to the mean FRET efficiency measured for AP1/AP1 (39.0% ± 16.9), but less variable (Additional file 1: Table S1). We also tested for SEP3 homomerization (Fig. 6B). As shown before, SEP3 can form homomeric complexes [33], although average BINDING (28.2% ± 9.7) was lower than detected for AP1/SEP3, but similar to AP1/AP1 (Additional file 1: Table S1). Hence, heteromer formation of AP1 with SEP3 appears to be preferential over the formation of individual homomeric complexes.

### AP3/PI heteromerization is dominant over AP3 or PI homomerization

**Fig. 7:**
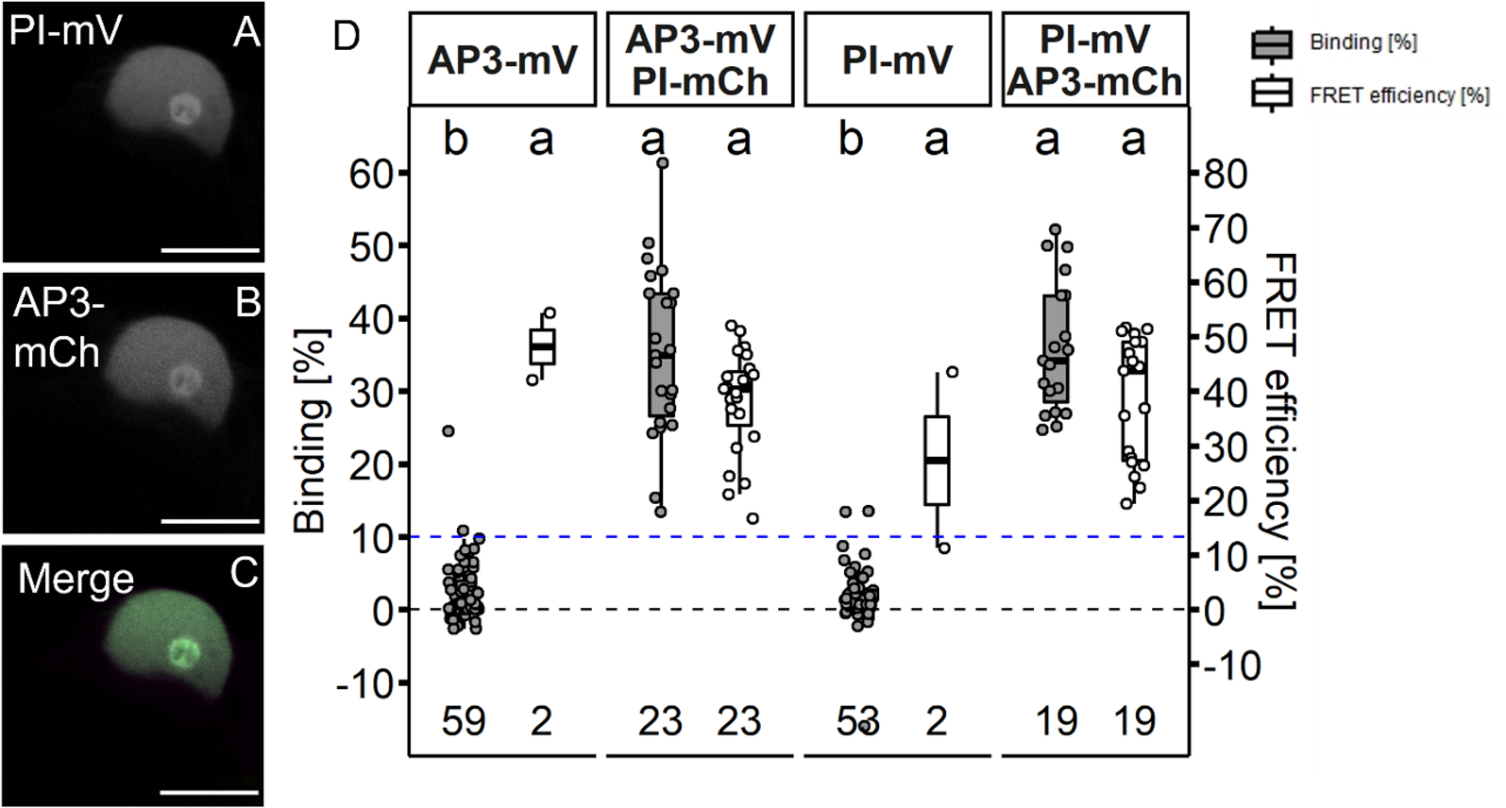
AP3 and PI homomerization in *N. benthamiana* leaf cells. AP3 and PI fused to the indicated FPs were expressed via the *UBQ10* promoter and imaged three days after infiltration. **A**–**C** Co-localisation of PI-mV and AP3-mCh in *N. benthamiana* leaf cells (**A** PI-mV signal. **B** AP3-mV signal. **C** Merged signal). Co-expression of AP3 with PI lead to an accumulation of both AP3 and PI in the nucleolus (for individual expressed AP3 and PI compare Fig. 3; Scalebars: **A**–**C** 10 µm). **D** BINDING [%] (grey) and FRET efficiencies [%] (white) for AP3-mV, AP3-mV PI-mCh, PI-mV and PI-mV AP3-mCh. Analysis was done as described in Fig. 5. Average BINDING between AP3 and PI was high in both measured directions (35.25% ± 11.65 for AP3/PI and 35.97% ± 8.99 for PI/AP3) with mean FRET Efficiencies of ~ 38%. Statistical groups were assigned after multiple comparison with Kruskal–Wallis and a Post hoc test using the criterium Fisher’s least significant difference (alpha parameter is 0.05) (Dashed blue line marks the BINDING cut-off of 10%; Number of repetitions are indicated below BINDING values and number of images with BINDING above 10% are indicated below the FRET efficiency values in the bottom of the plot)

Formation of AP3/PI heterodimers was previously characterised *in planta* by FRET-FLIM, BiFC and immunoprecipitation (IP) [18, 38]. We also detected localization to the nucleolus upon co-expression of the two proteins (Fig. 7A–C) as described [18, 29].

**Fig. 8:**
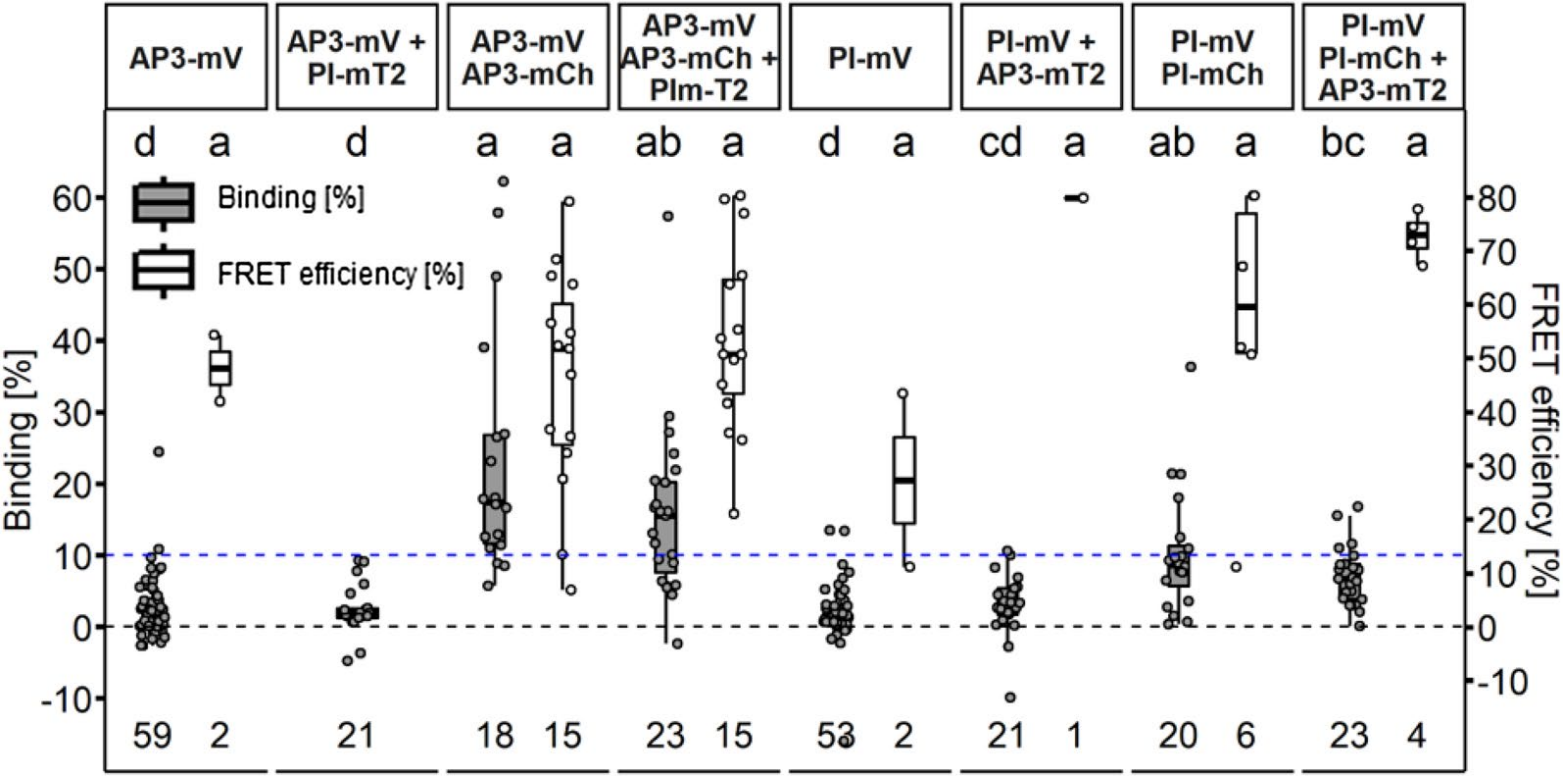
AP3 and PI protein homomerization in *N. benthamiana* leaf cells. BINDING (grey) and FRET efficiencies (white) for AP3-mV, AP3-mV + PI-mT2, AP3-mV AP3-mCh, AP3-mV AP3-mV-mCh + PI-mT2, PI-mV, PI-mV + AP3-mT2, PI-mV PI-mCh, PI-mV PI-mV-mCh + AP3-mT2. For each analysed nucleus average BINDING and a corresponding average lifetime of mV were fitted. Nucleoli were excluded from FLIM analysis. mT2 did not have an influence on mV lifetime as FRET can only occur from mT2 towards mV but not vice versa. Nuclei with average BINDING below 10% were excluded from FRET efficiency calculation. Statistical groups were assigned after multiple comparison with Kruskal–Wallis and a Post hoc test using the criterium Fisher’s least significant difference (alpha parameter is 0.05) (Dashed blue line marks the BINDING Cut-Off of 10%)

For this dimer, we determined high BINDING values (Fig. 7D, 35.3% ± 11.7 for AP3/PI and 36.0% ± 9.0 for PI/AP3), independent of the direction of the tested interaction. Heterodimerization of AP3 and PI is thought to be the evolutionary ancestral state and is necessary for DNA binding [47]. However, also homomeric interactions between AP3 or PI proteins have been reported in previous FRET-FLIM experiments [18]. In agreement with this, we could detect the formation of AP3/AP3 and PI/PI homomers, however with much lower affinities compared to AP3/PI heteromers (Fig. 8; 23.6% ± 17.2 and 10.2% ± 8.6 BINDING, respectively).

The addition of competitive PI to AP3/AP3 or AP3 to PI/PI samples, respectively, led to reduced, but statistically not significant average BINDING between AP3/AP3 or PI/PI when compared to samples without competitor (Fig. 8; 15.7% ± 12.04 and 7.0% ± 4.1 respectively).

### SEP3 is necessary for stable tetramer formation

**Fig. 9:**
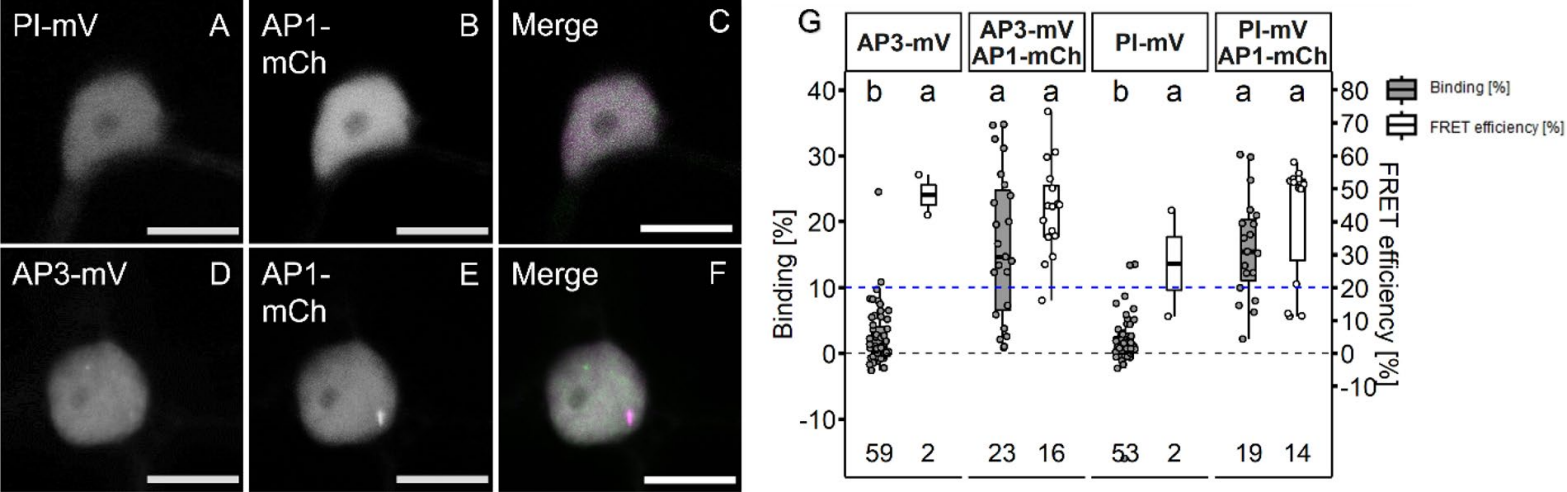
Interaction analysis between AP1 and AP3 or PI proteins in *N. benthamiana* leaf cells. A-C: Co-localisation of PI-mV an AP1-mCh in *N. benthamiana* leaf cells (**A** PI-mV signal. **B** AP1-mV signal. **C** Merged signal). **D**–**F** Co-localisation of AP3-mV an AP1-mCh in *N. benthamiana* leaf cells (**D** AP3-mV signal. **E** AP1-mV signal. **F** Merged signal). MADS-domain proteins fused to the respective FP were expressed in *N. benthamiana* leaf cells via the*UBQ10 promoter* (AP3 and PI) or the *XVE* <  < *oLexA-35S* estradiol inducible system (AP1). Nuclei were imaged 5–6 days after infiltration and expression of AP1 was induced one day prior to image acquisition. Co-expression of AP1 with AP3 or PI did not lead to a change of protein localisation. (Scalebars: **A**–**F** 10 µm) **G** BINDING [%] (grey) and FRET efficiencies [%] (white) for AP3-mV, AP3-mV AP1-mCh, PI-mV and PI-mV AP1-mCh. Analysis was done as described in Fig. 5. Both the AP1/AP3 and the AP1/PI heteromers displayed low average BINDING (16.46% ± 11.17 and 16.08% ± 7.77 respectively) and comparable FRET efficiencies of 43.61% ± 14.18 (AP1/AP3) and 41.54% ± 18.36. Statistical groups were assigned after multiple comparison with Kruskal–Wallis and a Post hoc test using the criterium Fisher’s least significant difference (alpha parameter is 0.05) (Dashed blue line marks the BINDING cut-off of 10%; Number of repetitions are indicated below BINDING values and number of images with BINDING above 10% are indicated below the FRET efficiency values in the bottom of the plot).

To date, interactions of AP1 with AP3 or PI could not be measured *in planta*. We therefore co-expressed AP3-mV and PI-mV with AP1-mCh transiently in the epidermis of *N. benthamiana* leaves (Fig. 9A–F).

**Fig. 10:**
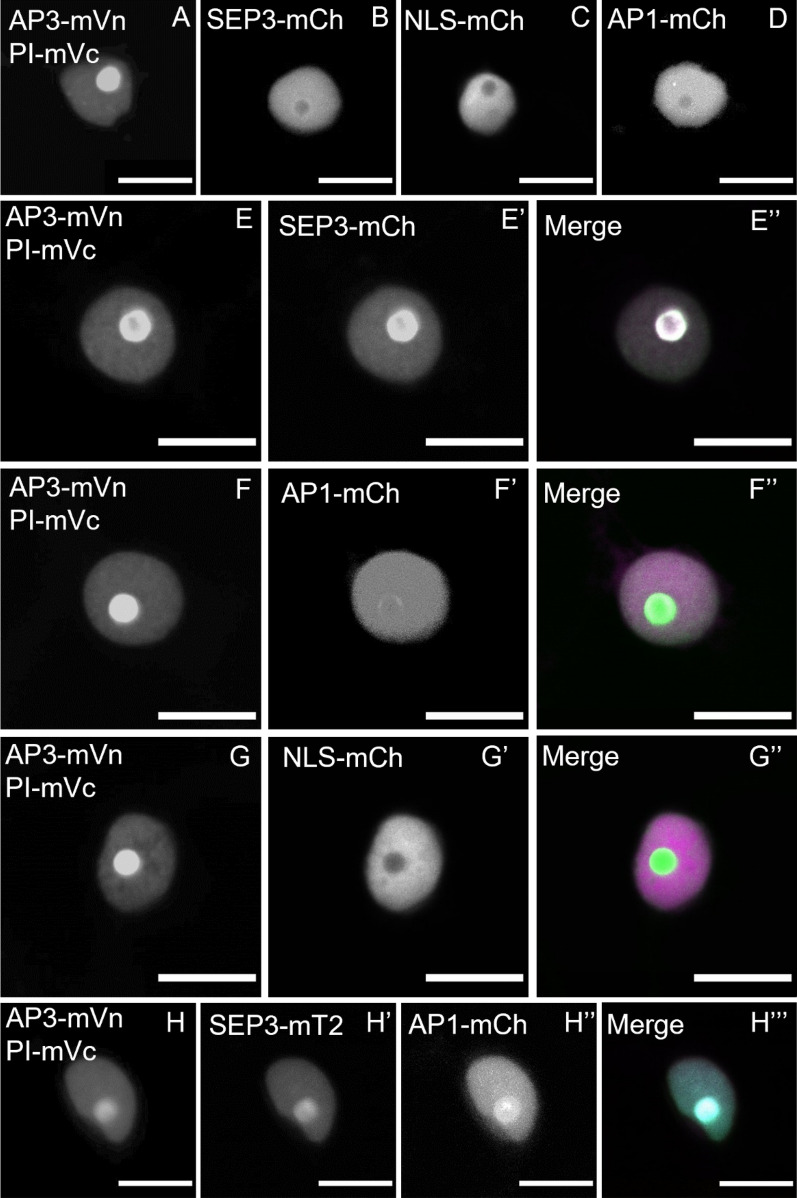
Localisation of co-expressed MADS-box proteins in *N. benthamiana* leaf cells. **A** Localisation of the AP3/PI heteromer visualized by BiFC. BiFC did not interfere with AP3/PI translocation to the nucleus or nucleolus. **B**–**D** Localisation of SEP3-mCh, NLS-mCh and AP1-mCh respectively. **E**–**E**’’: Co-expression of AP3/PI and SEP3 (**E** Signal from AP3/PI, E’: Signal from SEP3, **E**’’ Merged signal). SEP3 accumulated at the nucleolus and fully co-localized with AP3/PI. **F**–**F**’’ Co-expression of AP3/PI and AP1 (**F** Signal from AP3/PI, **F**’ Signal from AP1, **F**’’ Merged signal). AP1 weakly accumulated at in the presence of AP3/PI. **G**–**G**’’ Co-expression of AP3/PI and NLS-mCh (**G** Signal from AP3/PI, **G**’ Signal from NLS-mCh, **G**’’ Merged signal). NLS-mCh localisation did not change in the presence of AP3/PI. **H**–**H**’’’ Co-expression of AP3/PI with SEP3 and AP1 (**E** Signal from AP3/PI, **E**’ Signal from SEP3, **E**’’ Signal from AP1 **E**’’’ Merged signal). Expression of SEP3 with AP3/P and AP1 lead to strong re-localisation of AP1 to the nucleolus and full co-localisation of all four MADS-domain proteins. (Scale bars: 10 µm)

In this experiment, the cellular localization of the fusion proteins was identical when compared to those expressing the proteins individually but in contrast to the AP3/PI heteromer, there was no accumulation in the nucleolus of either AP1, AP3 or PI. Using FRET-FLIM, we determined BINDING value of ~ 16% for both AP1/AP3 and AP1/PI heteromers (Fig. 9G; 16.5% ± 11.2 and 16.1% ± 7.8 respectively), which, however, was lower than BINDING acquired for the AP3/PI or AP1/SEP3 heterodimers (Additional file 1: Table S1). Heteromeric complexes of AP1 and AP3 or PI therefore appear to be less stable perhaps the presence of an AP3/PI heterodimer or ability of tetramerization induced by other factors present are necessary to increase affinity. For example, the interaction of the AP3/PI heterodimer was enhanced by the addition of SEP3 in protoplast FRET-FLIM experiments [18]. Generic, two-fluorophore FRET-FLIM measurements only address the interaction between two partners. Hence, we combined FRET-FLIM with BiFC to analyse ternary or quaternary complex formation between the AP3/PI dimer and AP1 and SEP3 protein. One of the major downsides of BiFC is the high affinity of the two FP fragments for each other. As a result, the fragments may form stable fluorophores, although there may be no or only very weak interactions between the fused proteins of interest. As a proof of principle, AP3 and PI were tagged with the two FP fragments. Heteromerization of those two POIs is well characterized and appears to be essential for their stability, making them ideal partners for BiFC. mVenus was separated at amino acid residue 154. The N-terminal part (mVn) was tagged to AP3 and the C-terminal part (mVc) to PI. Fluorescence signal was detected in the nucleus of co-expressing epidermal cells and, as observed before, accumulated in the nucleolus, indicating no negative influence of the split mVenus on localization and complex formation (Fig. 10A).

**Fig. 11:**
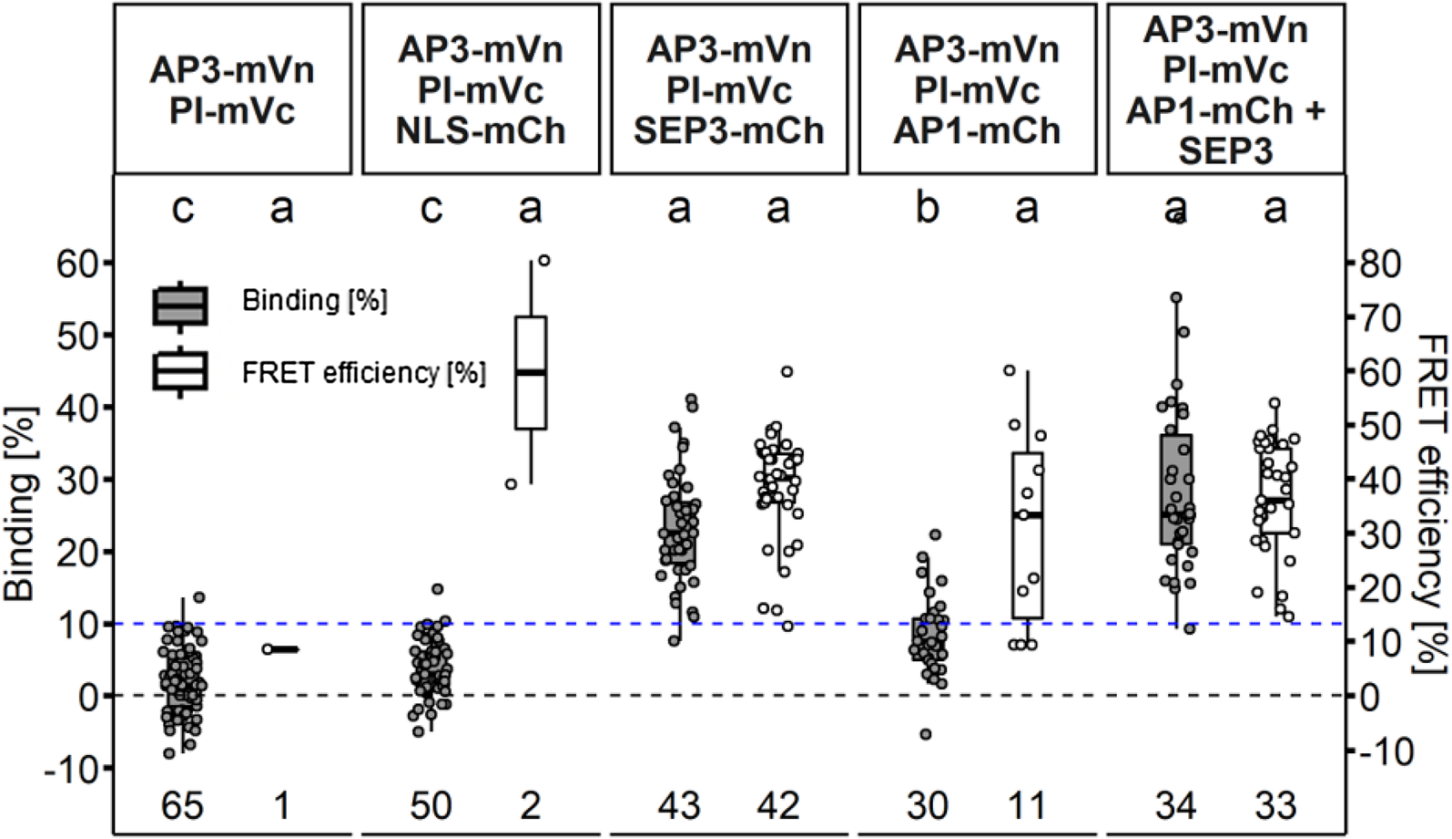
Larger complex formation between AP1, AP3, PI and SEP3 proteins in *N. benthamiana*. Higher order complex formation of MADS-box proteins was analysed by a combination of BiFC with FRET-FLIM. Complemented mV by the n-terminal and c-terminal part of mV tagged to AP3 and PI respectively served as the donor in FRET-FLIM experiments. All MADS-domain protein fused to FP were expressed via the UBQ10 promoter. Untagged SEP3 was expressed from the same T-DNA as AP1-mCh using the *XVE* <  < *oLexA-35S* estradiol inducible system. Images were acquired three days after infiltration and SEP3 was induced one day before imaging. BINDING [%] (grey) and FRET efficiencies [%] (white) for AP3-mVn PI-mVc, AP3-mVn PI-mVc NLS-mCh, AP3-mVn PI-mVc SEP3-mCh, AP3-mVn PI-mVc AP1-mCh and AP3-mVn PI-mVc AP1-mCh + SEP3. Analysis was done as described in Fig. 5. SEP3 together with AP3/PI displayed increased BINDING (23.21% ± 7.56) with a FRET efficiency of 38.57% ± 9.50. Affinity of AP1 for AP3/PI was low (8.40% ± 5.66 BINDING), but strongly increased in presence of SEP3 (28.80% ± 12.36 BINDING). Statistical groups were assigned after multiple comparison with Kruskal–Wallis and a Post hoc test using the criterium Fisher’s least significant difference (alpha parameter is 0.05) (Dashed blue line marks the BINDING cut-off of 10%; number of repetitions are indicated below BINDING values and number of images with BINDING above 10% are indicated below the FRET efficiency values in the bottom of the plot)

Because altered localization for the AP3/PI heteromer in the presence of SEP3 was previously reported [18], the individual expression of AP3-mVn/PI-mVc, AP1-mCh and SEP3-mCh was monitored before co-expression. NLS-mCh served as a non-interacting control for both comparisons of localization and later as negative control for the FRET-FLIM experiments. AP1-mCh, SEP3-mCh and NLS-mCh were mainly found in the nucleus but were absent from the nucleolus (Fig. 10B–D). Co-expression of SEP3 with AP3/PI had no effect on AP3/PI localization, contrary to the confocal laser scanning microscopy data from *Arabidopsis* protoplasts previously published [18]. Interestingly, we found the opposite effect: AP3/PI seems to promote accumulation of the other MADS-domain factors in the nucleolus. The previously only weakly nucleolus-associated SEP3 fully co-localized with AP3/PI and showed strong nucleolar accumulation (Fig. 10E–E’’). Although to a much weaker extend, this phenomenon could also be observed for AP1 (Fig. 10F–F’’). NLS-mCh had no influence on AP3/PI localisation and vice versa (Fig. 10G–G’’). Direct interactions between AP3-mVn/PI-mVc and AP1-mCh or SEP3-mCh were then analysed with FRET-FLIM. For the SEP3/AP3/PI combination, we measured increased BINDING values (Fig. 11; 23.2% ± 7.6), indicating ternary complexes or possible interaction between AP3/PI and SEP3/SEP3 dimers. In contrast, the combination of AP1/AP3/PI did not show high average BINDING values (Fig. 11; 8.4% ± 5.7), which were even lower compared to what we acquired before from the AP1/AP3 or AP1/PI combinations. We then tested whether co-expression of SEP3 could increase the affinity between AP1 and AP3/PI.

Indeed, additional SEP3 led to a stronger accumulation of AP1 in the nucleolus (Fig. 10H–H’’’) and strongly increased BINDING between AP1 and AP3/PI (Fig. 11; 28.8% ± 12.4).

To demonstrate the advantages OPA brings by separating BINDING from FRET efficiency, we included traditionally used average lifetimes, derived from bi-exponential fitting models, for the BiFC FRET-FLIM data (Additional file 1: Fig. S5). By using average lifetime analysis to evaluate the occurrence of FRET, we detected a significant influence of NLS-mCh on the AP3-mVn/PI-mVc donor lifetime, while we could not detect a significant difference between the NLS-mCh and AP1-mCh sample (Additional file 1: Fig. S5). In this case, average tau analysis suggests the occurrence of FRET in the negative control sample, while it could not detect FRET between AP3-mVn/PI-mVc and AP1-mCh. In contrast, OPA helps to better distinguish between interacting and non-interacting samples, as it shows no elevated BINDING between donor only and NLS-mCh, but significantly increased BINDING between AP3-mVn/PI-mVc and AP1-mCh.

Thus, by using OPA we were able to show that the predicted complex for petal specification in the FQM can form *in planta*. Furthermore, assembly of AP1 with AP3/PI proteins in higher order complexes is dependent on SEP3. Thus, we argue that the equilibria between dimer and tetramerization of AP1, AP3 and PI could indeed be controlled by the concentration of SEP proteins.

### FRET-FLIM could not detect MADS-domain protein interactions in young floral buds

Different MADS-domain protein di- and tetramers are thought to form in a whorl- specific manner in the developing tissue, however, *ex-situ* experiments reach their limits in the spatial and temporal resolution of complex formation. Therefore, it is of special interest to study such complexes predicted directly in developing floral meristems (FM). In 2012, Smaczniak and colleagues isolated MADS-domain protein complexes by immunoprecipitation and characterised them using LC–MS/MS [38]. While their data suggest that the proposed MADS-domain protein complexes form in developing flowers, these experiments lacked tissue-specific and cellular resolution. Additionally, in the same report, BiFC experiments were used to detect interaction between AG/SEP3, AP1/SEP3 and AP3/PI. Compared to BiFC, FRET assays have the advantage of having higher spatial resolution and, more importantly, are more specific, as complementing FP fragments show a tendency for self-assembly resulting in false positive interactions [13, 37]. FLIM in contrast to intensity- or spectral-based FRET methods, is more gentle on cells and tissues due to lower required laser intensity and is generally considered the more accurate method to detect FRET [10, 26, 27, 35, 40, 41].

**Fig. 12:**
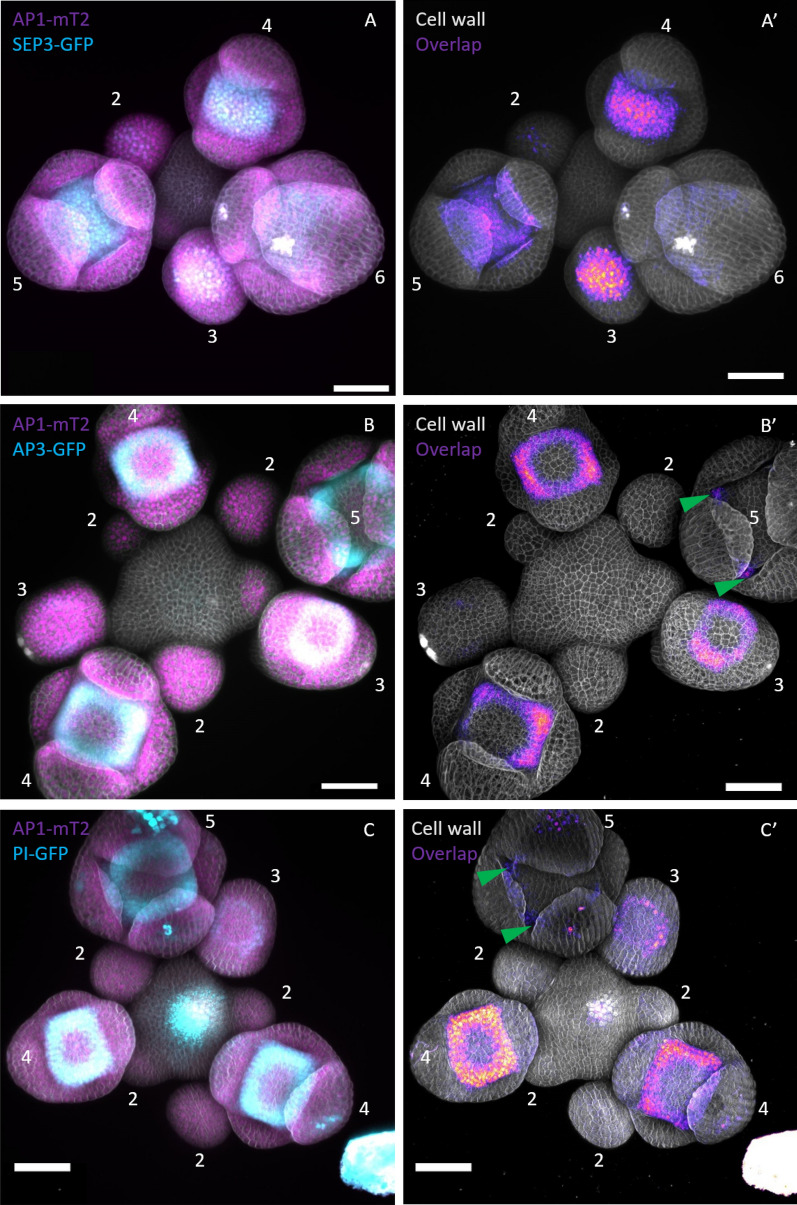
Co-expression of AP1-mT2 with GFP reporeter of SEP3, AP3 and PI. **A**–**C** Z-stacks of AP1-mT2 (red) SEP3-GFP (green), AP1-mT2 (red) APP3-GFP (green) and AP1-mT2 (red) PI-GFP (green). **A**’–**C**’ signal overlap was calculated in FIJI [19] using the Image calculator tool and were displayed with the “fire” color scale (low signal: purple; high signal: yellow). AP1 and SEP3 expression overlaps in few cells of late stage 2 floral buds and most cells of the dome shaped floral meristem in stage 3, stage 4 and stage 5 flowers. Overlaped expression of AP1 with AP3 or PI is first visibale in stage 3 floral buds. In stage 4 flowers AP1 and AP3 or PI proteins show an overlapping expression in a ringformed pattern while in stage 5 flowers, overlapping expression was restricted to petal initiation sites. Numbers indicate floral stage as previously defined (Smyth, Bowman, and Meyerowitz 1990). Scale bars: 50 µm

Therefore, we here investigated interactions of AP1, SEP3, PI and AP3 in early-stage flowers using FRET-FLIM. Previously, GFP reporter of these MADS-domain proteins were generated and characterised [6, 48] and we aimed to use these as donor lines in FRET-FLIM experiments. To ensure high saturation of donor proteins with acceptor, we chose AP1 as acceptor, because it displayed the highest fluorescence signal among the MADS-box proteins we aimed to analyse. A ~ 3 kb promoter region upstream of the AP1 start codon was used to drive expression of AP1-mT2, AP1-mV or AP1-mCh fusion proteins. Constructs were transformed in *ap1-1* mutant plants using the floral dip method [51]. To analyse interactions between AP3 and PI we also generated a PI-mCh reporter with ~ 2 kb gPI fragment as promoter. The proPI::PI-mCh construct was transformed into wild-type plants and then crossed into the *pi-1* mutant background for complementation assays. All FP fusion constructs were able to rescue the respective stamen and/or petal deficiency of the *pi-1* or *ap1-1* mutant (Additional file 1: Fig. S9). The expression pattern of the GFP reporter as well as the here established AP1-FP and PI-mCh lines, analysis by confocal laser scanning microscopy, were in agreement with the expression pattern previously described [6, 32, 46, 48] for AP1, SEP3, AP3 and PI (Additional file 1: Fig. S7, S9). For co-localisation analysis of AP1 with SEP3, AP3 or PI we crossed an AP1-mT2 reporter with the SEP3, AP3 or PI GFP reporter and imaged the F1 progeny. We observed overlapping expression of SEP3 and AP1 in few cells of stage 2 floral buds and in the FM of stage 3, 4 and 5 flowers (Fig. 12A–A’).

**Fig. 13:**
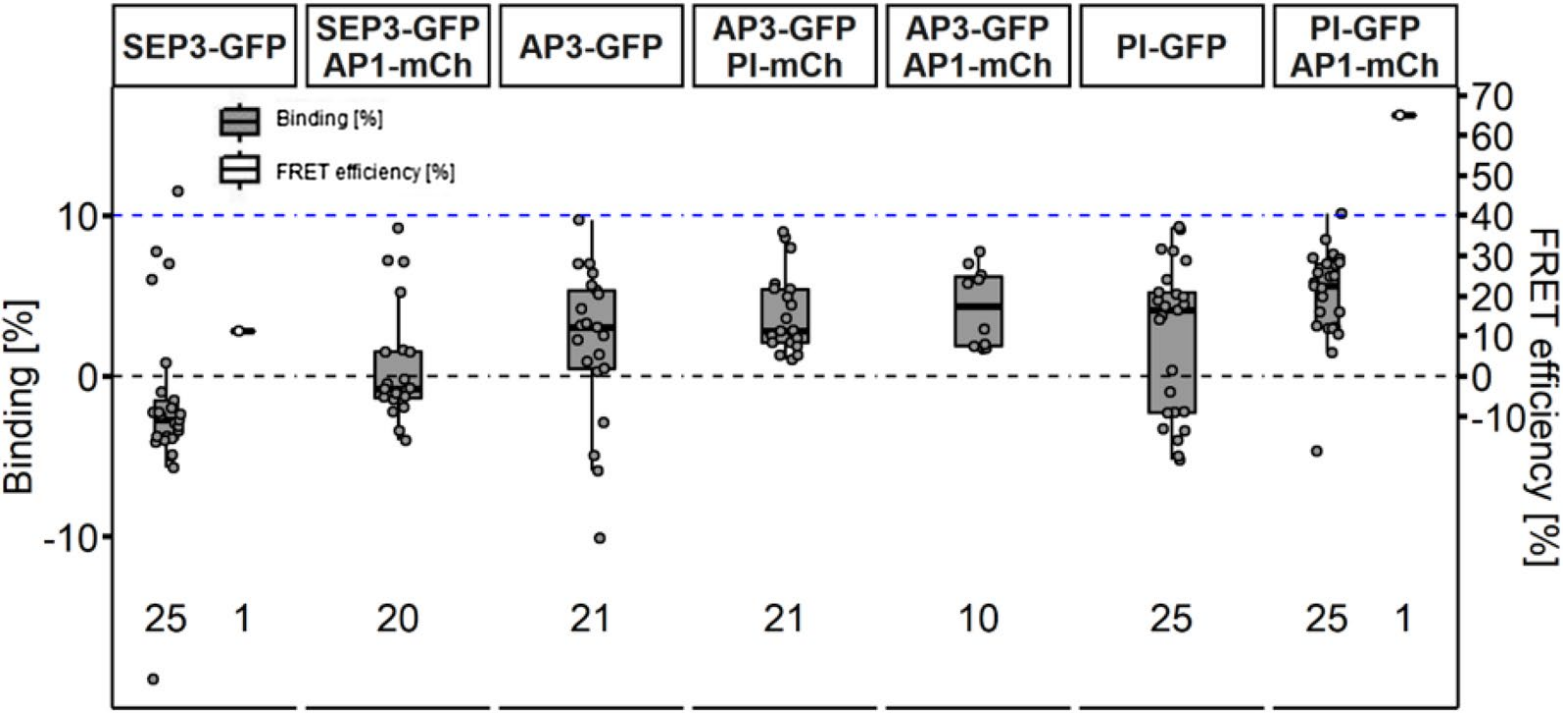
No detectable interaction between MADS-domain proteins with FRET-FLIM in Arabidopsis GFP/mCh reporter. FLIM experiments were performed in stable *A. thaliana* lines. MADS-box proteins fused with the indicated FP were expressed by their endogenous promoter. Donor only images were acquired in homozygous GFP reporter lines. For the FRET samples, GFP lines were crossed with the respective mCherry reporter and FLIM images were acquired in resulting F1 plants. Each data point was calculated from a respective FLIM image containing several nuclei. Only nuclei containing both donor and acceptor were considered in FLIM image analysis of FRET samples. BINDING [%] (grey) and FRET efficiencies [%] (white) for SEP3-GFP, SEP3-GFP AP1-mCh, AP3-GFP, AP3-GFP PI-mCh, AP3-GFP AP1-mCh, PI-GFP and PI-GFP AP1-mCh. No increased BINDING above the 10% cut-off in FRET samples was detectable for any of the tested combinations. Statistical groups were assigned after multiple comparison with Kruskal–Wallis and a Post hoc test using the criterium Fisher’s least significant difference (alpha parameter is 0.05) (Dashed blue line marks the BINDING Cut-Off of 10%; Number of repetitions are indicated below BINDING values and number of images with BINDING above 10% are indicated below the FRET efficiency values in the bottom of the plot)

AP3 and PI co-localised in stage 3, 4 and 5 flowers (Fig. 12B–B’, C–C’). In stage 3 and 4 buds, overlap between AP1 and AP3/PI proteins was detected in the ring formed expression pattern, characteristic for AP3 and PI, while in stage 5 flowers overlap was restricted to petal initiation sites (Fig. 12B’ and C’; green arrows). For FRET-FLIM experiments, SEP3, AP3 and PI GFP reporter were crossed with a AP1-mCh or PI-mCh reporter line and lifetime images were acquired in the F1 progeny. Because of low signal from SEP3-, AP3- and PI-GFP we slightly increased the laser power of the 485 nm laser (from 1 to 1.4 µW at the objective). In deeper tissues of stage 5 flowers and petal initiation sites signal intensity was strongly reduced and we could not acquire suitable amounts of photons. We therefore restricted FRET-FLIM experiments to stage 3 and 4 flowers. To avoid autofluorescence, ROIs were used to select several nuclei per image and plastids with low lifetimes were excluded. Surprisingly, we could not detect any increase in BINDING above background in AP1/AP3, AP1/SEP3 PI/SEP3 or AP3/PI expressing plants (Fig. 13; Additional file 1: Table S2).

BINDING values of nuclei containing both donor and acceptor molecules did no increase and were comparable to donor only nuclei in our analysis.

### AP1 forms homomers in young floral organs

**Fig. 14:**
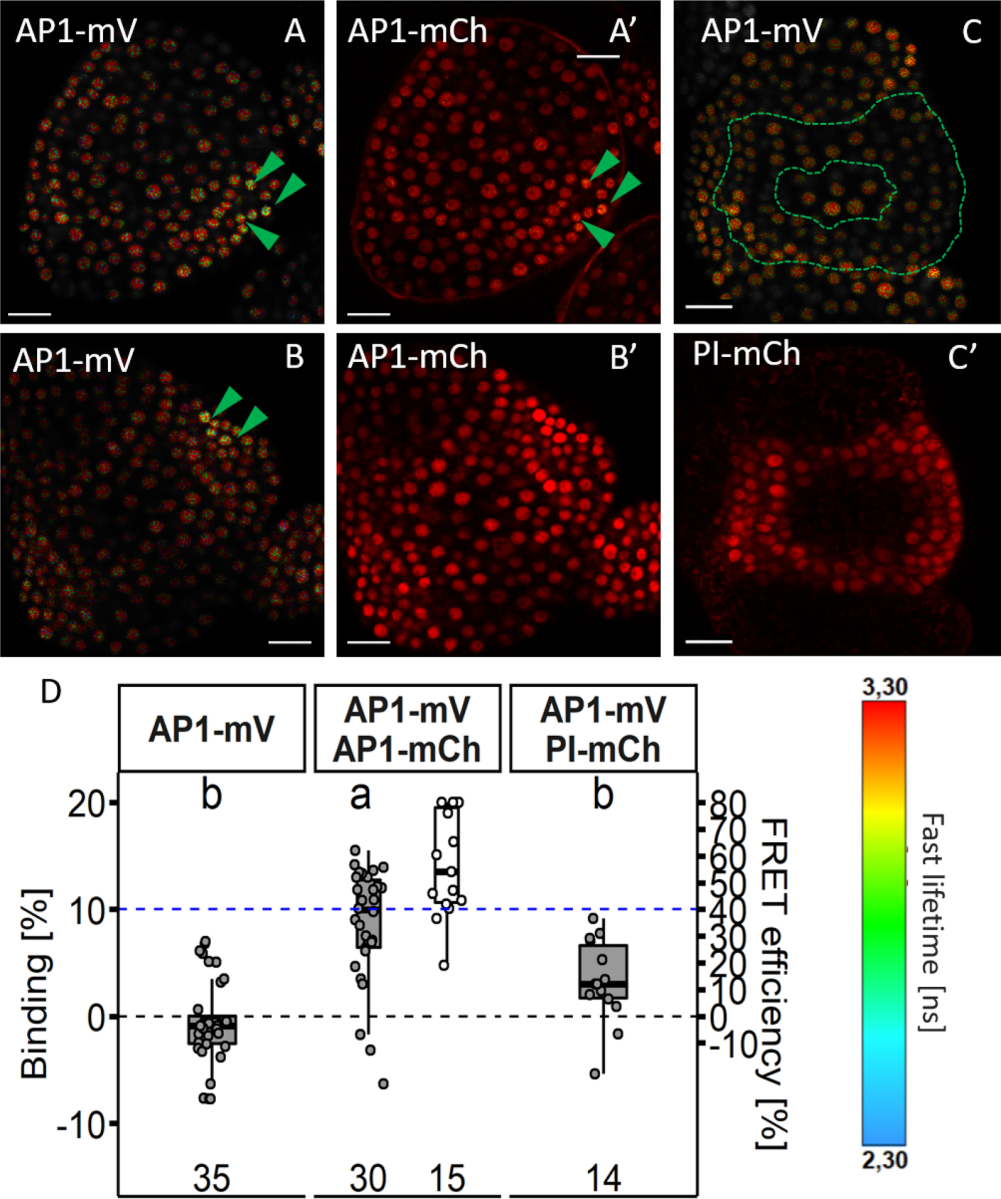
AP1 homomer formation and no interaction between AP1 and PI. **A**, **B** Fast Lifetime images of AP1-mV in presence of AP1-mCh (depicted in **A**’–**B**’, Zoom2). Green arrows highlight nuclei with decreased lifetime. **C** Lifetime images of AP1-mV in presence of PI-mCh (depicted in **C**–**C**’, Zoom2). Dashed green line highlights region where AP1 and PI co-localise. No difference in lifetime between nuclei with or without acceptor could be observed. **D** BINDING [%] (grey) and FRET efficiencies [%] (white) for AP1-mV, AP1-mV AP1-mCh and AP1-mV PI-mCh. (Dashed blue line marks the BINDING cut-off of 10%; number of repetitions are indicated in the bottom of the plot). Data points were calculated from a respective FLIM image containing several nuclei (Zoom8 or Zoom2), but only nuclei containing both donor and acceptor were considered in FLIM image analysis of FRET samples. In half the images of AP1-mV AP1-mCh samples increased BINDING above the 10% Cut-off was measured (mean BINDING of 8.59% ± 5.37and mean FRET efficiency of 56.67% ± 19.52), while in the AP1-mV PI-mCh samples no BINDING above the 10% Cut-off could be detected (3.28% ± 3.91). Statistical groups were assigned after multiple comparison with Kruskal–Wallis and a post-hoc test using the criterium Fisher’s least significant difference (alpha parameter is 0.01). Scale bars: 40 µm

Since photon counts in the *Arabidopsis* experiments were lower compared to experiments in *N. benthamiana* (Additional file 1: Fig. S8), we wondered whether photon counts in *Arabidopsis* were just too low to detect FRET with our fitting model. Because AP1 expression is stronger compared to AP3, PI and SEP3 we tried AP1-mV as the donor, crossed it with our AP1-mCh or PI-mCh reporter line (Additional file 1: Fig. S9) and acquired lifetime images in the F1 progeny. Nuclei expressing PI-mCh as acceptor did no display decreased average donor lifetime and we could not detect significantly increased BINDING values (Additional file 1: Table S2; Fig. 14C–C’; D), comparable to the data we measured for PI-GFP AP1-mCh. In AP1-mV AP1-mCh expressing plants we detected nuclei with significantly increased BINDING slightly above 10% (Fig. 14; Additional file 1: Table S2), indicating homomer formation in these cells. Interestingly, we observed these interactions only in few cells with high protein concentration in young sepals (Fig. 14A–A’; B–B’).

## Discussion

FRET is a commonly used tool to investigate PPI in vivo. A frequently used technique to measure FRET is time domain FLIM. FLIM generates quantitative data providing information on protein affinities and spatial arrangement of the protein complexes under investigation. However, in the field of plant science, commonly lifetime or average amplitude weighted lifetime has been used to evaluate interactions, leading to the loss of valuable information [7, 10, 17, 18, 45].

Additionally, when evaluating our negative control and donor only samples, we found that for most samples, we could reliably fit an additional lifetime, but with a low relative amplitude. Lifetimes without context of their relative amplitudes should therefore be avoided to evaluate FRET. Instead, the possibility of resolving for BINDING and FRET efficiency from a complex decay behaviour as demonstrated here not only provides more information about complex formation, but also acts as a better measure of the presence of FRET and as an intrinsic control for FRET efficiencies.

Based on the results outlined above, we propose to use BINDING, which is derived from the amplitudes of the decay fractions and FRET efficiency, as a measure for protein affinity and proximity within the forming complexes respectively. This differentiation between BINDING and FRET efficiency to describe FRET usually requires donor-only decays with a monoexponentially decaying behaviour. The OPA method described here is specifically tailored for bi-exponentially decaying donors. It comes as a special script, which can be implemented into SymPhoTime 64 software and can be obtained by PicoQuant on request. Additionally, the underlying model is described in more detail in the methods section, allowing the integration of OPA in other publicly available FLIM analysis scripts.

By investigating MADS-domain protein interactions as a proof of principle, we showed that OPA can serve to acquire BINDING and FRET efficiencies for bi-exponentially decaying donors. Additionally, OPA was able to determine BINDING to judge the occurrence of FRETin developing flowers, where protein expression levels from the native promoters are low.

In vivo interactions of MADS-domain proteins were previously mainly characterised by BiFC, intensity-based FRET or FLIM-FRET [14, 17, 18, 28, 38, 45]. BiFC and intensity-based methods come with the disadvantage of being prone to false positives. In addition, intensity-based FRET analyses are rather unsuitable for measuring interactions in living meristems or flowers due to the high laser radiation required and the associated photodamage of living tissues and fluorophores. In the FLIM-FRET experiments previously conducted, only lifetimes were used to assess FRET [18, 45]. While average lifetime analysis is a suitable tool to display PPI, it has difficulties to evaluate information of differences in causality for these interactions, as both increased affinity or conformational changes within the forming complexes can result in the reduction of average lifetime. In contrast, OPA can dissolve differences in affinity and protein proximity by separating BINDING and FRET efficiency.

Therefore, we repeated previously reported hetero-dimeric interactions of AP1 with SEP3 and AP3 with PI *in planta* [18], by also involving corresponding amplitudes of lifetimes, not only giving us additional information about the affinity of the proteins to each other, but also more confident data (Figs. 6, 7). We observed stable homomeric complexes formed by AP1 or SEP3 and stable interaction between AP3/PI (Figs. 5, 6). In line with the idea that homomer formation of AP3 or PI proteins is an ancestral state and the fact that DNA binding of either AP3 or PI requires AP3/PI heteromerization [47], we did not observe stable interaction between AP3/AP3 nor PI/PI (Fig. 8). Interaction of AP1 with AP3 or PI proteins is one of the major interactions proposed by the FQM model but was not yet precisely analysed *in planta.* While average lifetime analysis failed to detect significant FRET between AP3/PI and AP1, OPA revealed low affinity between AP1 and AP3, PI or both (Figs. 9, 11; Additional file 1: Fig. S5). Addition of SEP3 boosted the binding between AP3/PI and AP1 while proximity did not significantly change (Fig. 11, Additional file 1: Fig. S5), suggesting that quaternary complex formation with high affinity between AP1, AP3 and PI relied on the presence of SEP3 protein (Figs. 10, 11), This heavily supports the role of SEP proteins as a “glue” between MADS-domain proteins [18]. Similar to observations made with MADS-domain proteins from lily (*Lilium longiflorum*) [28], mean FRET-efficiencies of stable complexes were usually within the same range (Additional file 1: Table S1, Fig. S6), suggesting comparable proximity between the respective C-termini in the individual complexes.

Because tetramer composition is supposed to be of particular importance for the activity for MADS-domain proteins involved in floral organ specification, approaches which only look at two proteins at a time come to their limit for studying tetramer stoichiometry. The combination of BiFC with FRET-FLIM employed in the present study, is a first step towards reliably analysing binding specifications of the MADS-domain proteins and their tetrameric organization in vivo.

While informative, the characterization of PPIs in heterologous systems or in in vitro assays cannot fully replace an analysis in the cells or tissues in which the proteins under study are normally expressed. We therefore also attempted to assess different floral MADS-domain protein complexes in early-stage *Arabidopsis* flowers. In these experiments, we could not observe heteromeric interactions between AP1, SEP3, AP3 and PI, even though they have been detected by FRET in heterologous systems, or by BiFC and pull-down assays in *Arabidopsis* [18, 38]. It is possible that low endogenous expression levels and/or the presence of competing interaction partners, which are lacking in heterologous systems, could lead to a strong reduction of the FRET fraction, making it more difficult to detect interactions in FLIM assays. Future optimization of FRET set ups, and/or the use of improved spectroscopic methods, will likely be necessary to detect and quantify multimeric MADS-domain protein complex formation in vivo.

## Conclusion

By re-assessing MADS-domain protein interactions, we here demonstrate that OPA can be used to extract both protein affinities and spatial information from FRET samples with bi-exponential donor decays in transient expression models like *N. benthamiana* but also in developing flowers of *Arabidopsis*.

While in the past mostly in vitro approaches for the analysis of MADS-domain protein interactions dominated the field, *in planta* approaches will be needed for a better characterization of putative interactions and to distinguish relevant complexes in a developmental context. Although experiments in *Arabidopsis* ultimately display the natural mechanisms of organ specification most accurately, experiments in transient plant systems such as *Arabidopsis* protoplasts or *N. benthamiana* leaf cells, already allow the observation of proteins in a more native environment than in vitro studies.

Transient expression reflects only a small part of the reality in which MADS-domain protein complexes can form. Of greater interest, though, is complex formation in the native environment during flowering. We tried to address this problem, but low expression levels or high donor–acceptor ratios made it difficult to observe any interaction. As FRET-FLIM is considered as a precise technique to quantify protein–protein interaction, our results raise the question whether previous data from BiFC experiments, which are more susceptible to false negative results and ignore transient interactions, can reliably be trusted. With our novel FLIM FRET analysis method we can more precisely dissect interactions to give important insights about protein affinity and complex formation in living tissues.

## Supplementary Information

**Additional file 1: Fig. S1.** FLIM analysis of AP1-mV NLS-mCh and AP1-mV Ap1-mCh in *N. benthamiana* leaf cells. FLIM experiments were performed in *N. benthamiana* leaf epidermis cells. Fusion proteins were expressed from the *UBQ10* promoter and imaged 3–4 days after infiltration. BINDING [%] (grey) and FRET efficiencies [%] (white) for AP1-mV, AP1-mV NLS-mCh and AP1-mV AP1-mCh. Depicted are the same data as in Fig. 1, but FRET efficiencies with BINDING below 10% were not excluded. FRET efficiencies of AP1-mV and AP1mV AP1-mV-NLS-mCh samples have a higher variance compared to the AP1-mV AP1-mCh sample. When the value for BINDING was below 10%, FRET efficiencies showed a higher tendency for values close to the limits of the fitting model (10% and 80% FRET efficiencies). (Dashed blue line marks the BINDING Cut-Off of 10%; Number of repetitions are indicated below BINDING values and number of images with BINDING above 10% are indicated below the FRET efficiency values in the bottom of the plot). **Fig. S2.** Comparison of the One pattern analysis and a mono exponential Donor decay model. BINDING values for AP1-mV samples, fitted with the One pattern analysis or an analysis which assumes a mono exponentially decaying donor model. For the One pattern analysis, three lifetime components were fitted (see methods for details), and for the analysis which assumes a mono exponential decaying donor, two lifetime components were fitted. Due to the influence of the secondary shorter mV lifetime, the analysis assuming a monoexponentially decaying donor results in increased BINDING of more than 10% in almost one third of the images indicating false positive FRET. In contrast, using the One pattern analysis, most acquired BINDING values are below the 10% limit and are less variable. **Fig. S3.** Change in BINDING and FRET efficiency acquired in the same cell before and after acceptor bleaching. FLIM experiments were performed in *N. benthamiana* leaf cells. Fusion proteins were expressed via te *UBQ10* promoter and images were acquired 3 days after infiltration. After the first time series, photobleaching of mCherry was performed with a 561nm laser at 100 % for ninety frames, followed b a second time series. Analysis was done as described in Fig. 2. Binding between AP1-mV and AP1-mCh was not detectable anymore after acceptor bleaching. (Dashed blue line marks the BINDING cut-off of 10%; Number of repetitions are indicated below BINDING values and number of images with BINDING above 10% are indicated below the FRET efficiency values in the bottom of the plot). **Fig. S4.** Dependence of BINDING on acceptor concentration. **B** BINDING values calculated for AP1-mV AP1-mCh, and corresponding photons detected in the acceptor channel. BINDING correlates with the Acceptor concentration. **Fig. S5**. Standard fluorescence lifetime analysis of AP3-mVn PI-mVc samples. Donor only decays were fitted using a monoexponential decay model while decays from donor + acceptor samples were fitted using a biexponential decay model. The non-FRET sample, containing NLS-mCh, shows a significant reduction in fluorescence lifetime compared to the donor only sample (AP3-mVn PI-mVc). In contrast with the OPA, standard lifetime analysis does not reveal interaction between the NLS-mCh containing sample and the AP1-mCh containing sample. Only for the SEP3-mCh and AP1-mCh + SEP3 containing samples a significant reduction in fluorescence lifetime compared to the NLS-mCh containing sample was detectable. Statistical groups were assigned after multiple comparison with Kruskal-Wallis and a Post hoc test using the criterium Fisher’s least significant difference (alpha parameter is 0.05) (Number of repetitions are indicated in the bottom of the plot). **Fig. S6.** Average BINDING and FRET efficiencies of all FRET and negative control samples. Interacting samples are labelled in green and non-interacting samples are labelled in magenta. Occurrence of interaction was judged by a significant increase in BINDING based on Kruskal-Wallis and a Post hoc test using the criterium Fisher’s least significant difference (alpha parameter is 0.05). Error bars indicate the standard error. **Fig. S7**. MADS-domain protein reporters. Expression pattern of AP1-mT2 in *ap1-1* (**A**), SEP3-GFP (**B**), PI-GFP in *pi-1* (**C**) and AP3-GFP in *ap3-3* (**D**). Expression of AP1 starts in stage 2 floral buds. In stage 3 and stage 4 buds AP1 is braoldy expressed. Expression starts to become restricted to sepals and petal initiation sites stating in stage 5 flowers. Weak expressionin of SEP3 starts in late stage 2 buds. In lates stage buds SEP3 is is mexpressed in cells of the third and fourth whorl. AP3 and PI became visible in early stage 3 florla buds and are expressed in circular pattern in the second and third whorl of stage 4 and stage 5 flowers. In stage 6 flowers expression of AP3 or PI proteins is restricted to developing stamen and peatl initiation sites. Numbers indicate floral stage as previously defined (Smyth, Bowman, and Meyerowitz 1990). (Z-stacks, Scale Bars: 50 µm). **Fig. S8**. Photons in donor channel in different donor only samples. Number of photons was measured in ROIs marking several nuclei in Arabidopsis samples or one nucleus in *N. benthamiana* samples. Nucleoli of *N. benthamiana* nuclei were excluded from analysis. Counts in Arabidopsis GFP reporter lines of AP3, PI and SEP3 were lower compared to counts in the AP1-mV reporter or in *N. benthamiana* samples. **Fig. S9**. Co-expression of AP1 with PI and mutant complementation of *ap1-1* and *pi-1* and. **A**–**A**’’: Z-stack of AP1-mV signal, AP1-mCh signal and Merged signals respectivly. **B**–**B**’’: Z-stack of AP1-mV signal, PI-mCh signal and Merged signals respectivly. (Scale Bars: 50 µm). **C**–**F** Inflocrescences of *ap1-1* (**C**–**E**) or *pi-1* (**F**) mutants complemented with AP1-mCh (**C**), AP1-mV (**D**), AP1-mT2 (**E**) and PI-mCh (**F**). Indicated MADS-domain proteins, tagged with FPs, were expressed via their endogenous promoter. All fusion proteins rescued the organ deficient phenotype of the mutants in 4 (*ap1-1*) or 2 (*pi-1*) independent lines. **Table S1.** Summary of the FRET-FLIM measurements for the investigation of MADS-domain protein interactions in *N. benthamiana*. Mean, standard deviation (SD) and standard error (SE) of FRET efficiencies were calculated after removing values with BINDING < 10%. **Table S2.** Summary of the FRET-FLIM measurements for the investigation of MADS-domain protein interactions in *Arabidopsis*. Mean, standard deviation (SD) and standard error (SE) of FRET efficiencies were calculated after removing values with BINDING < 10%. **Table S3. **Plasmids used, but not constructed during this study. **Table S4.** Plasmids used from the GreenGate kit. **Table S5. **“Entry” plasmids generated in this study. The list contains all “entry” plasmids which were used for the construction of plant expression plasmids. Plasmids were cloned by restriction ligation using *BsaI*. Inserts were amplified with the respective primers from the according templates and cloned in the appropriate backbone. **Table S6. **Plant expression plasmids constructed in this study. Plasmids were used for stable *A. thaliana* transformation or transient transformation of *N. benthamiana*. Construction of the plasmids was achieved by the GreenGate method using the appropriate Inserts and assemble them in the respective Backbone. **Table S7. **Oligonucleotides used in this study.

## Data Availability

The datasets used and/or analysed during the current study are available from the corresponding author upon request.
