# Supplementary material for: One pattern analysis (OPA) for the quantitative determination of protein interactions in plant cells
Source: Plant Methods. 2023 Jul 28;19:73. doi: 10.1186/s13007-023-01049-3 (PMC10375638; doi:10.1186/s13007-023-01049-3)
Supplement: Supplementary file 1 — Additional file 1: Fig. S1. FLIM analysis of AP1-mV NLS-mCh and AP1-mV Ap1-mCh in N. benthamiana leaf cells. FLIM experiments were performed in N. benthamiana leaf epidermis cells. Fusion proteins were expressed from the UBQ10 promoter and imaged 3–4 days after infiltration. BINDING [%] (grey) and FRET efficiencies [%] (white) for AP1-mV, AP1-mV NLS-mCh and AP1-mV AP1-mCh. Depicted are the same data as in Fig. 1, but FRET efficiencies with BINDING below 10% were not excluded. FRET efficiencies of AP1-mV and AP1mV AP1-mV-NLS-mCh samples have a higher variance compared to the AP1-mV AP1-mCh sample. When the value for BINDING was below 10%, FRET efficiencies showed a higher tendency for values close to the limits of the fitting model (10% and 80% FRET efficiencies). (Dashed blue line marks the BINDING Cut-Off of 10%; Number of repetitions are indicated below BINDING values and number of images with BINDING above 10% are indicated below the FRET efficiency values in the bottom of the plot). Fig. S2. Comparison of the One pattern analysis and a mono exponential Donor decay model. BINDING values for AP1-mV samples, fitted with the One pattern analysis or an analysis which assumes a mono exponentially decaying donor model. For the One pattern analysis, three lifetime components were fitted (see methods for details), and for the analysis which assumes a mono exponential decaying donor, two lifetime components were fitted. Due to the influence of the secondary shorter mV lifetime, the analysis assuming a monoexponentially decaying donor results in increased BINDING of more than 10% in almost one third of the images indicating false positive FRET. In contrast, using the One pattern analysis, most acquired BINDING values are below the 10% limit and are less variable. Fig. S3. Change in BINDING and FRET efficiency acquired in the same cell before and after acceptor bleaching. FLIM experiments were performed in N. benthamiana leaf cells. Fusion proteins were expressed via te UBQ [file 13007_2023_1049_MOESM1_ESM.docx]

# Additional file information

## Supporting Figures


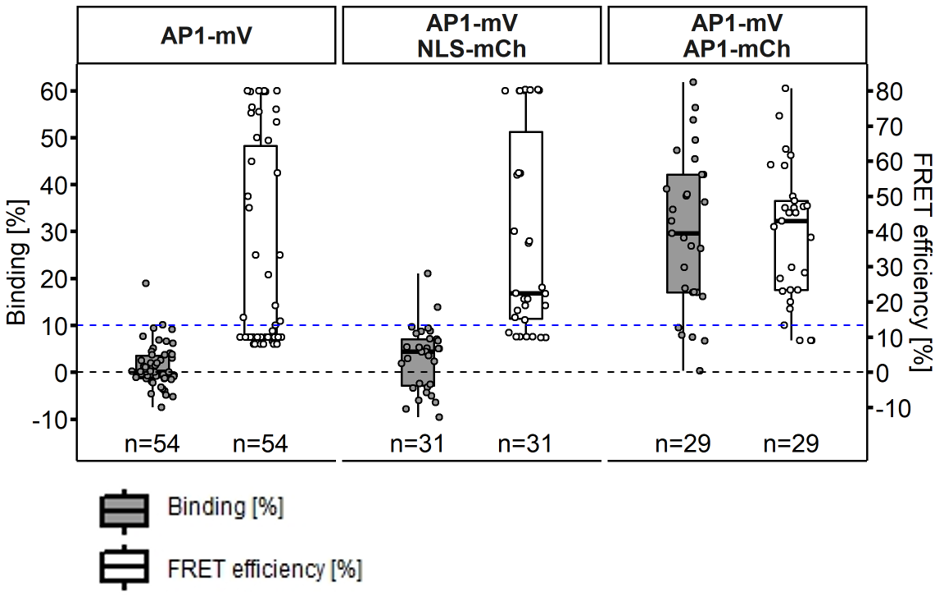


**Fig. S1: FLIM analysis of AP1-mV NLS-mCh and AP1-mV Ap1-mCh in *N. benthamiana* leaf cells.**

FLIM experiments were performed in *N. benthamiana* leaf epidermis cells. Fusion proteins were expressed from the *UBQ10* promoter and imaged 3-4 days after infiltration. BINDING [%] (grey) and FRET efficiencies [%] (white) for AP1-mV, AP1-mV NLS-mCh and AP1-mV AP1-mCh. Depicted are the same data as in Figure 1, but FRET efficiencies with BINDING below 10% were not excluded. FRET efficiencies of AP1-mV and AP1mV AP1-mV-NLS-mCh samples have a higher variance compared to the AP1-mV AP1-mCh sample. When the value for BINDING was below 10%, FRET efficiencies showed a higher tendency for values close to the limits of the fitting model (10% and 80% FRET efficiencies). (Dashed blue line marks the BINDING Cut-Off of 10%; Number of repetitions are indicated below BINDING values and number of images with BINDING above 10% are indicated below the FRET efficiency values in the bottom of the plot).


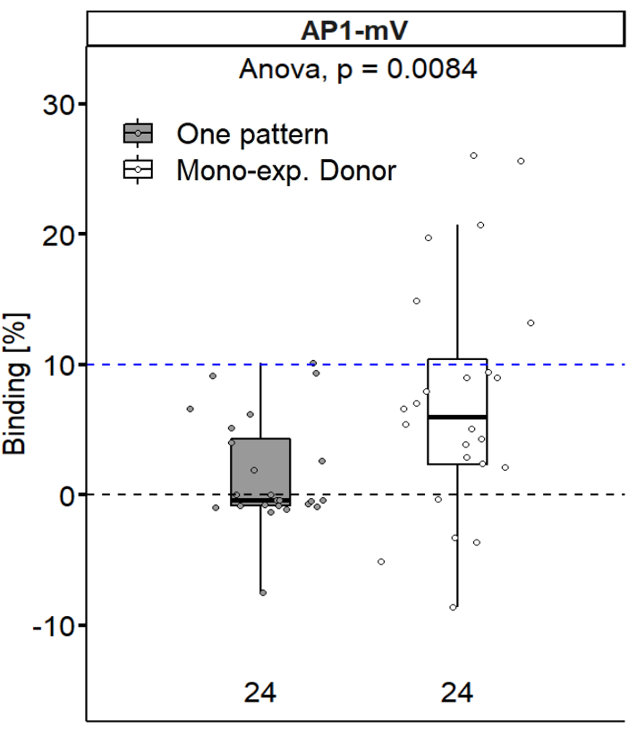


**Fig. S2: Comparison of the One pattern analysis and a mono exponential Donor decay model.**

BINDING values for AP1-mV samples, fitted with the One pattern analysis or an analysis which assumes a mono exponentially decaying donor model. For the One pattern analysis, three lifetime components were fitted (see methods for details), and for the analysis which assumes a mono exponential decaying donor, two lifetime components were fitted. Due to the influence of the secondary shorter mV lifetime, the analysis assuming a monoexponentially decaying donor results in increased BINDING of more than 10% in almost one third of the images indicating false positive FRET. In contrast, using the One pattern analysis, most acquired BINDING values are below the 10% limit and are less variable.

**
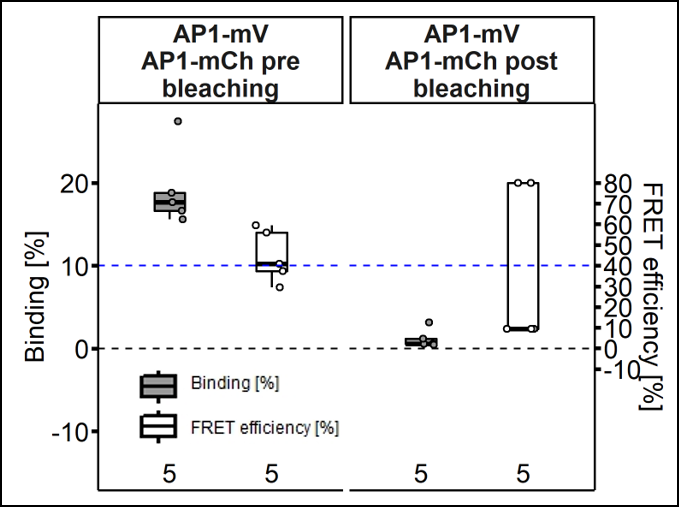
**

**Fig. S3: Change in BINDING and FRET efficiency acquired in the same cell before and after acceptor bleaching.**

FLIM experiments were performed in *N. benthamiana* leaf cells. Fusion proteins were expressed via te *UBQ10* promoter and images were acquired 3 days after infiltration. After the first time series, photobleaching of mCherry was performed with a 561nm laser at 100 % for ninety frames, followed b a second time series. Analysis was done as described in Fig. 2. Binding between AP1-mV and AP1-mCh was not detectable anymore after acceptor bleaching. (Dashed blue line marks the BINDING cut-off of 10%; Number of repetitions are indicated below BINDING values and number of images with BINDING above 10% are indicated below the FRET efficiency values in the bottom of the plot).


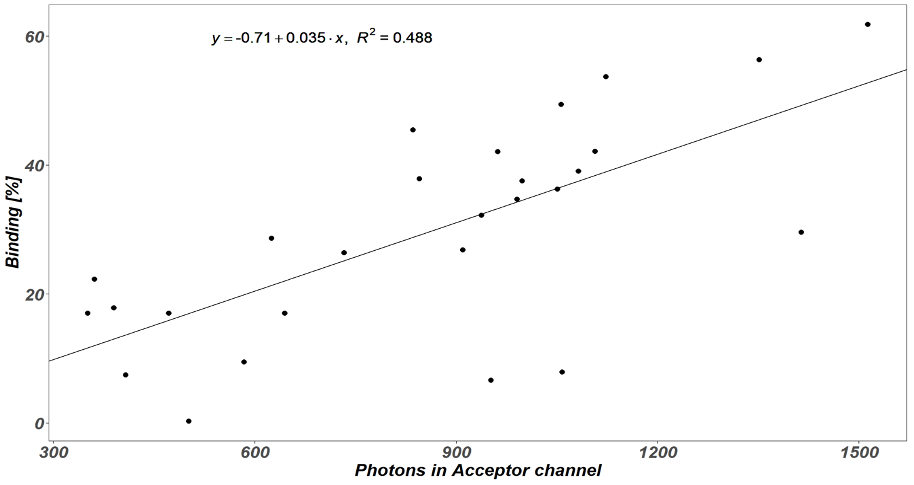


**Fig. S4: Dependence of BINDING on acceptor concentration.**
B: BINDING values calculated for AP1-mV Ap1-mCh, and corresponding photons detected in the acceptor channel. BINDING correlates with the Acceptor concentration.


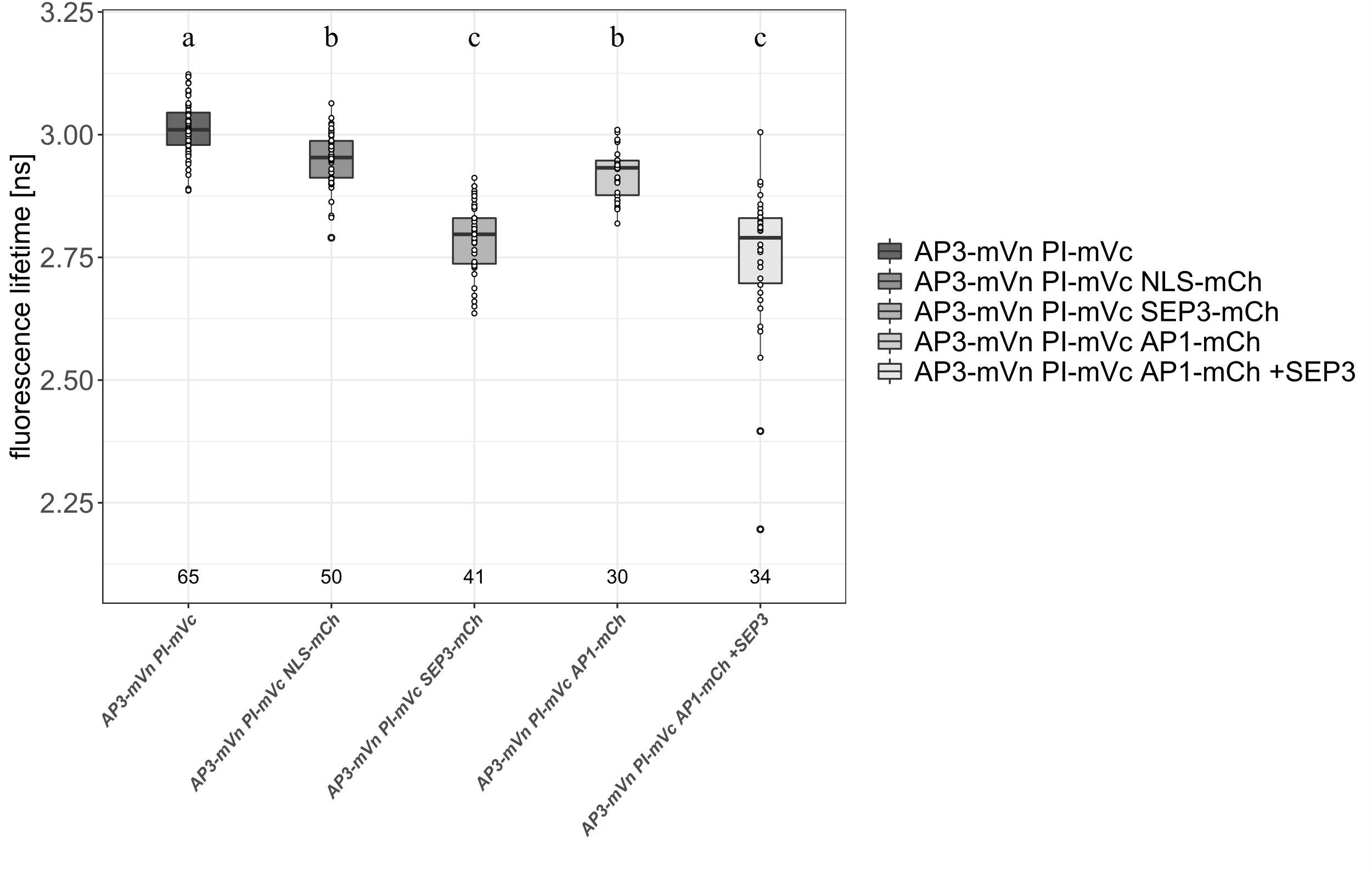


**Fig. S5 Standard fluorescence lifetime analysis of AP3-mVn PI-mVc samples.**

Donor only decays were fitted using a monoexponential decay model while decays from donor + acceptor samples were fitted using a biexponential decay model. The non-FRET sample, containing NLS-mCh, shows a significant reduction in fluorescence lifetime compared to the donor only sample (AP3-mVn PI-mVc). In contrast with the OPA, standard lifetime analysis does not reveal interaction between the NLS-mCh containing sample and the AP1-mCh containing sample. Only for the SEP3-mCh and AP1-mCh + SEP3 containing samples a significant reduction in fluorescence lifetime compared to the NLS-mCh containing sample was detectable. Statistical groups were assigned after multiple comparison with Kruskal-Wallis and a Post hoc test using the criterium Fisher’s least significant difference (alpha parameter is 0.05) (Number of repetitions are indicated in the bottom of the plot).


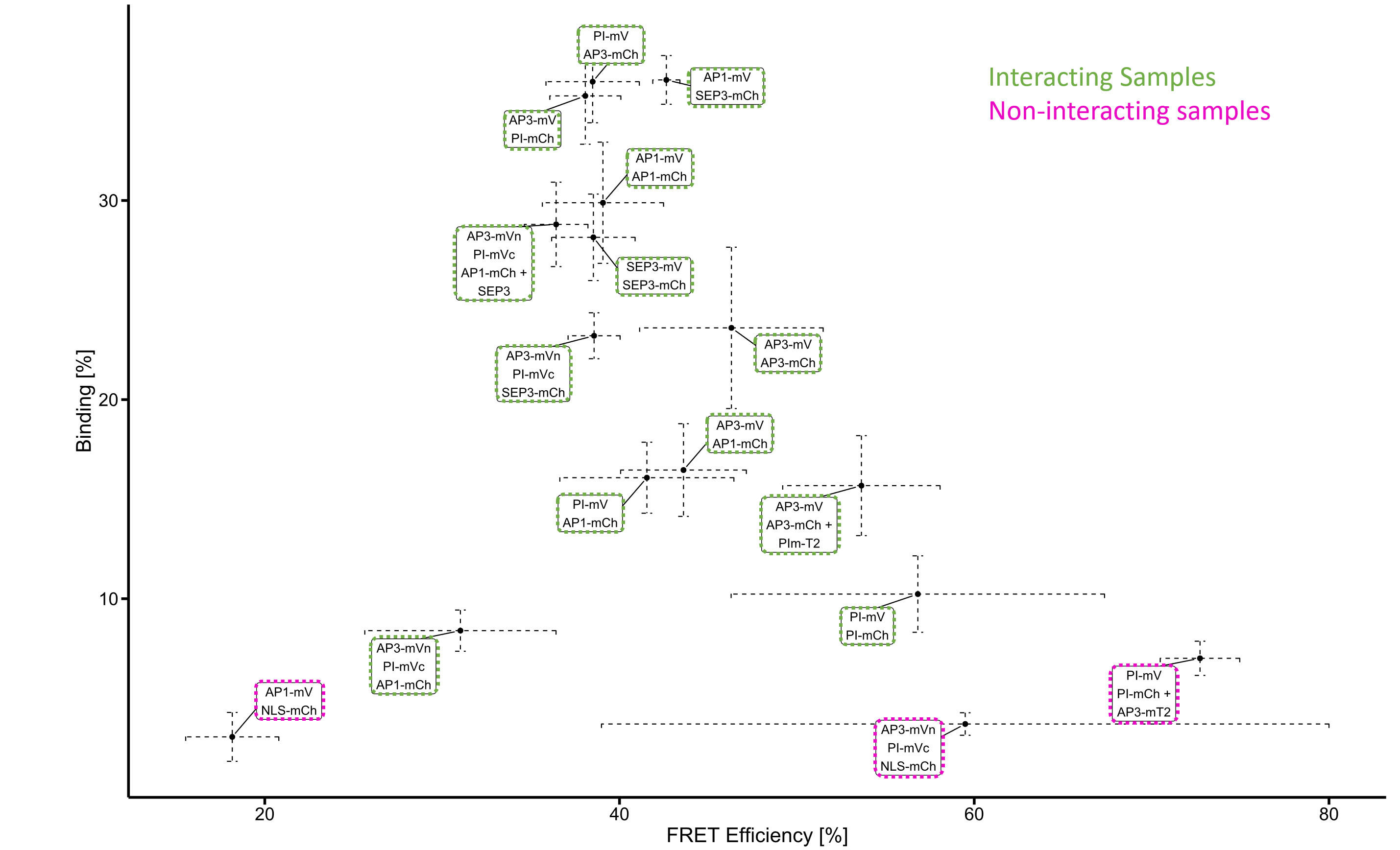


Fig. S6: Average BINDING and FRET efficiencies of all FRET and negative control samples. Interacting samples are labelled in green and non-interacting samples are labelled in magenta. Occurrence of interaction was judged by a significant increase in BINDING based on Kruskal-Wallis and a Post hoc test using the criterium Fisher’s least significant difference (alpha parameter is 0.05). Error bars indicate the standard error.


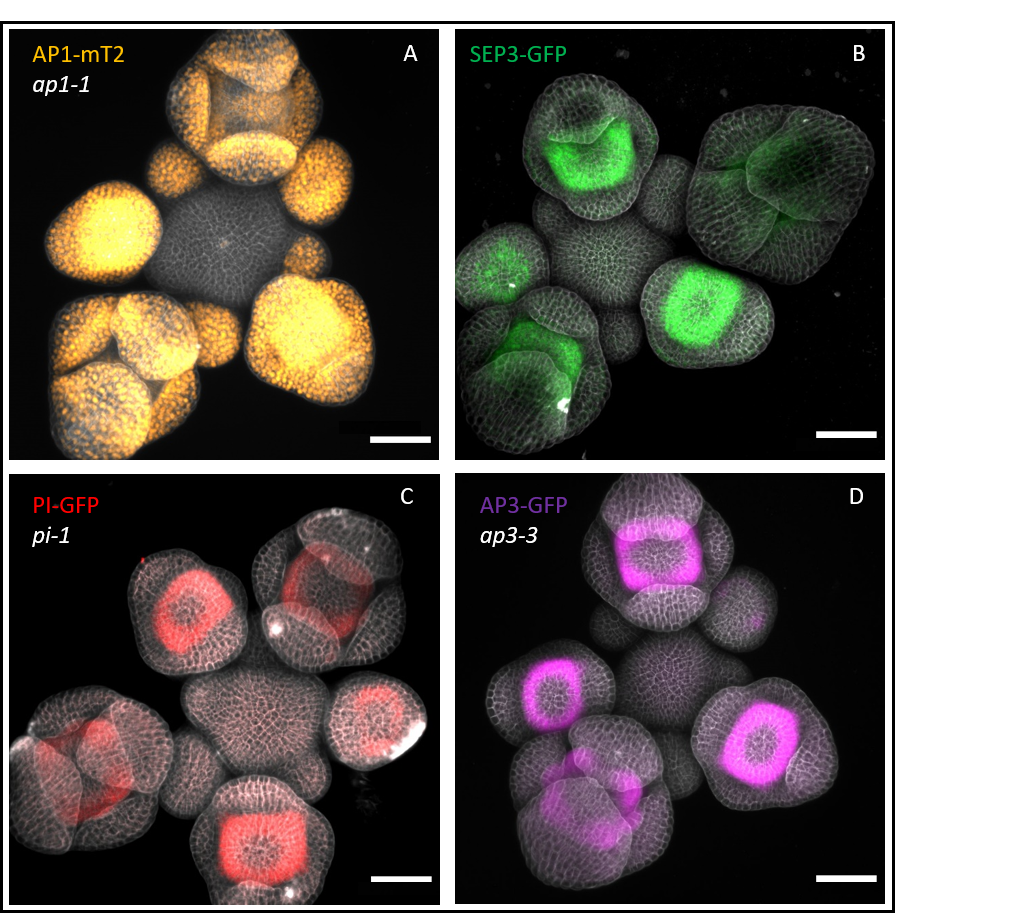


**Fig. S7: MADS-domain protein reporters.**

Expression pattern of AP1-mT2 in *ap1-1* (A), SEP3-GFP (B), PI-GFP in *pi-1* (C) and AP3-GFP in *ap3-3* (D). Expression of AP1 starts in stage 2 floral buds. In stage 3 and stage 4 buds AP1 is braoldy expressed. Expression starts to become restricted to sepals and petal initiation sites stating in stage 5 flowers. Weak expressionin of SEP3 starts in late stage 2 buds. In lates stage buds SEP3 is is mexpressed in cells of the third and fourth whorl. AP3 and PI became visible in early stage 3 florla buds and are expressed in circular pattern in the second and third whorl of stage 4 and stage 5 flowers. In stage 6 flowers expression of AP3 or PI proteins is restricted to developing stamen and peatl initiation sites. Numbers indicate floral stage as previously defined (Smyth, Bowman, and Meyerowitz 1990). (Z-stacks, Scale Bars: 50 µm).


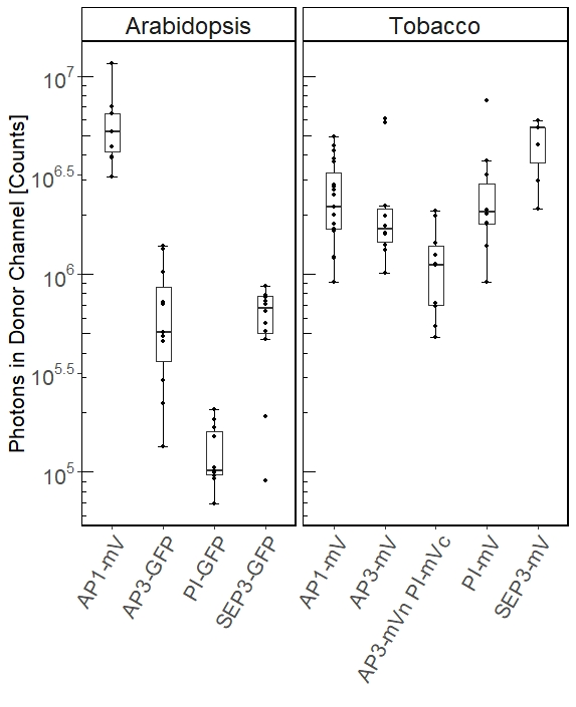


**Fig. S8: Photons in donor channel in different donor only samples.**

Number of photons was measured in ROIs marking several nuclei in Arabidopsis samples or one nucleus in *N. benthamiana* samples. Nucleoli of *N. benthamiana* nuclei were excluded from analysis. Counts in Arabidopsis GFP reporter lines of AP3, PI and SEP3 were lower compared to counts in the AP1-mV reporter or in *N. benthamiana* samples.


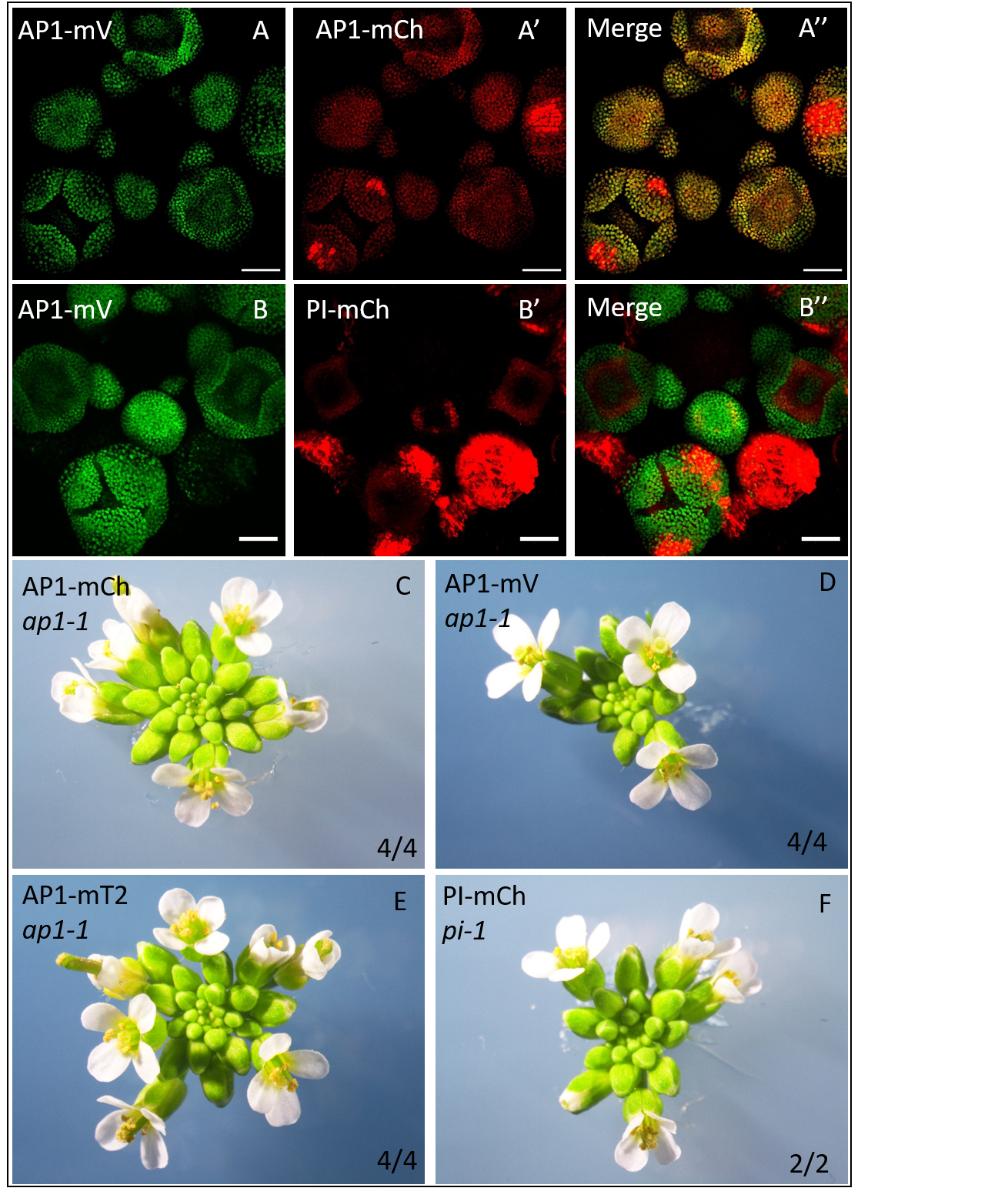


**Fig. S9: Co-expression of AP1 with PI and mutant complementation of *ap1-1* and *pi-1* and.**

A-A’’: Z-stack of AP1-mV signal, AP1-mCh signal and Merged signals respectivly. B-B’’: Z-stack of AP1-mV signal, PI-mCh signal and Merged signals respectivly. (Scale Bars: 50 µm). C-F: Inflocrescences of *ap1-1* (C-E) or *pi-1* (F) mutants complemented with AP1-mCh (C), AP1-mV (D), AP1-mT2 (E) and PI-mCh (F). Indicated MADS-domain proteins, tagged with FPs, were expressed via their endogenous promoter. All fusion proteins rescued the organ deficient phenotype of the mutants in 4 (*ap1-1*) or 2 (*pi-1*) independent lines.

## Supporting Tables

**Tab. S1. Summary of the FRET-FLIM measurements for the investigation of MADS-domain protein interactions in *N. benthamiana*.** Mean, standard deviation (SD) and standard error (SE) of FRET efficiencies were calculated after removing values with BINDING < 10%.

| **Sample** | **mean BINDING [%]** | **SD BINDING [%]** | **SE BINDING [%]** | **mean FRET efficiency [%]** | **SD FRET efficiency [%]** | **SE FRET efficiency [%]** |
| --- | --- | --- | --- | --- | --- | --- |
| AP1-mV | 1.23 | 4.48 | 0.61 | 10.83 | 1.18 | 0.83 |
| AP1-mV AP1-mCh | 29.89 | 16.39 | 3.04 | 38.98 | 16.89 | 3.45 |
| AP1-mV AP1-mCh post bleaching | 1.17 | 1.15 | 0.51 | NA | NA | NA |
| AP1-mV AP1-mCh pre bleaching | 19.21 | 4.74 | 2.12 | 44.61 | 12.67 | 5.67 |
| AP1-mV NLS-mCh | 3.06 | 6.84 | 1.23 | 18.17 | 3.71 | 2.63 |
| AP1-mV SEP3-mCh | 36.06 | 10.01 | 1.22 | 42.64 | 6.26 | 0.77 |
| AP3-mV | 2.41 | 4.33 | 0.56 | 48.13 | 8.67 | 6.13 |
| AP3-mV + PI-mT2 | 2.45 | 3.46 | 0.76 | NA | NA | NA |
| AP3-mV AP1-mCh | 16.46 | 11.17 | 2.33 | 43.61 | 14.18 | 3.54 |
| AP3-mV AP3-mCh | 23.60 | 17.19 | 4.05 | 46.10 | 20.45 | 5.28 |
| AP3-mV AP3-mCh + PIm-T2 | 15.68 | 12.04 | 2.51 | 53.66 | 17.21 | 4.44 |
| AP3-mV PI-mCh | 35.25 | 11.65 | 2.43 | 38.07 | 9.61 | 2.00 |
| AP3-mVn PI-mVc | 1.83 | 4.48 | 0.56 | 8.50 | NA | NA |
| AP3-mVn PI-mVc AP1-mCh | 8.40 | 5.66 | 1.03 | 30.26 | 18.98 | 5.72 |
| AP3-mVn PI-mVc AP1-mCh + SEP3 | 28.80 | 12.36 | 2.12 | 36.43 | 10.31 | 1.79 |
| AP3-mVn PI-mVc NLS-mCh | 3.71 | 4.00 | 0.57 | 59.63 | 29.20 | 20.64 |
| AP3-mVn PI-mVc SEP3-mCh | 23.21 | 7.56 | 1.15 | 38.57 | 9.50 | 1.47 |
| PI-mV | 1,86 | 4,03 | 0,55 | 34,19 | 30,63 | 4,21 |
| PI-mV + AP3-mT2 | 3.16 | 4.41 | 0.96 | 79.80 | NA | NA |
| PI-mV AP1-mCh | 16.08 | 7.77 | 1.78 | 41.54 | 18.36 | 4.91 |
| PI-mV AP3-mCh | 35.97 | 8.99 | 2.06 | 38.49 | 11.47 | 2.63 |
| PI-mV PI-mCh | 10.23 | 8.59 | 1.92 | 56.91 | 25.88 | 10.57 |
| PI-mV PImCh + AP3-mT2 | 7.01 | 4.12 | 0.86 | 72.73 | 4.49 | 2.24 |
| SEP3-mV | 1.89 | 4.14 | 0.90 | 10.00 | 0.00 | 0.00 |
| SEP3-mV SEP3-mCh | 28.15 | 9.71 | 2.17 | 38.53 | 10.53 | 2.35 |

**Tab. S2. Summary of the FRET-FLIM measurements for the investigation of MADS-domain protein interactions in *Arabidopsis*.** Mean, standard deviation (SD) and standard error (SE) of FRET efficiencies were calculated after removing values with BINDING < 10%.

| **Sample** | **mean BINDING [%]** | **SD BINDING [%]** | **SE BINDING [%]** | **mean FRET efficiency [%]** | **SD FRET efficiency [%]** | **SE FRET efficiency [%]** |
| --- | --- | --- | --- | --- | --- | --- |
| AP1-mV | -0.54 | 3.91 | 0.66 | NA | NA | NA |
| AP1-mV AP1-mCh | 8.59 | 5.37 | 0.98 | 56.67 | 19.52 | 5.04 |
| AP1-mV PI-mCh | 3.28%± | 3.91 | 1.04 | NA | NA | NA |
| AP3-GFP | 2.08 | 4.79 | 1.05 | NA | NA | NA |
| AP3-GFP AP1-mCh | 4.3 | 2.47 | 0.78 | NA | NA | NA |
| AP3-GFP PI-mCh | 3.83 | 2.41 | 0.53 | NA | NA | NA |
| PI-GFP | 2.36 | 4.7 | 0.94 | NA | NA | NA |
| PI-GFP AP1-mCh | 4.99 | 2.95 | 0.59 | 64.91 | NA | NA |
| SEP3-GFP | -1.88 | 5.67 | 1.13 | 11.24 | NA | NA |
| SEP3-GFP AP1-mCh | 0.67 | 3.68 | 0.82 | NA | NA | NA |

**Tab. S3. Plasmids used, but not constructed during this study.**

| **ID** | **Insert** | **Overhangs** | **Bacterial resistance** | **Received from** |
| --- | --- | --- | --- | --- |
| pRD43 | mVenus | C-tag | Amp r | Rebecca Burkart |
| pRD53 | mCherry | C-tag | Amp r | Rebecca Burkart |
| pBLAD011 | mTurquoise2 | C-tag | Amp r | Andrea Bleckmann |
| pGD283 | XVE destination vector | - | Spec r | Grégoire Denay |
| pBLAA001 | pLexA-mini35S | - | Amp r | Andrea Bleckmann |

**Tab. S4. Plasmids used from the GreenGate kit**

| **ID** | **Insert** | **Overhangs** | **Bacterial resistance** |
| --- | --- | --- | --- |
| pGGA000 | Plant promoter Entry vector | A-B | Amp r |
| pGGC000 | Coding sequence Entry vector | C-D | Amp r |
| pGGD000 | C-tag | D-E | Amp r |
| pGGE000 | Plant terminator | E-F | Amp r |
| pGGZ001 | Destination vector | A-G | Spec r |
| pGGA002 | AP3 (APETALA3; internal *Bsa*I site removed) promoter | A-B | Amp r |
| pGGA006 | UBQ10 (UBIQUITIN10) promoter | A-B | Amp r |
| pGGD007 | Linker-NLS | D-E | Amp r |
| pGGB002 | Ω (Omega- element) | B-C | Amp r |
| pGGB003 | B-dummy (default random sequence) | B-C | Amp r |
| pGGD002 | D-dummy (default random sequence) | D-E | Amp r |
| pGGE001 | RBCS terminator (from pea) | E-F | Amp r |
| pGGE009 | UBQ10 terminator | E-F | Amp r |
| pGGF003 | pMAS:D-AlaR:tMAS | F-G | Amp r |
| pGGF005 | pUBQ10:HygrR:tOCS | F-G | Amp r |
| pGGF007 | pNOS:KanR:tNOS | F-G | Amp r |
| pGGF008 | pNOS:BastaR (chi sequence removed):tNOS | F-G | Amp r |

**Tab. S5. "Entry" plasmids generated in this study.** The list contains all “entry” plasmids which were used for the construction of plant expression plasmids. Plasmids were cloned by restriction ligation using *BsaI*. Inserts were amplified with the respective primers from the according templates and cloned in the appropriate backbone.

| **ID** | **Insert** | **Backbone** | **Oligos** | **Template** |
| --- | --- | --- | --- | --- |
| pJM1/tOCS | tOCS | pGGE000 | oJM47/oJM48 | pGGF005 |
| pJM5/cdsAP3 | *APETALA3* genomic region without stop | pGGC000 | oJM5/oJM6 | L-*er* gDNA |
| pJM8/pAP1 | 3kb *APETALA1* promoter region | pGGA000 | oJM9/oJM10 | L-*er* gDNA |
| pJM9/cdsAP1 | *APETALA1* genomic region without stop | pGGC000 | oJM7/oJM8 | L-*er* gDNA |
| pJM11/pPI | 2kb *PISTILLATA* promoter region | pGGA000 | oJM13/oJM14 | L-*er* gDNA |
| pJM12/cdsPI | *PISTILLATA* genomic region without stop | pGGC000 | oJM11/oJM12 | L-*er* gDNA |
| pJM81/mVenusSplit1 | nTerminus of mVenus (aa 1-154) | pGGD000 | oJM108/oJM109 | pRD43 |
| pJM82/mVenusSplit2 | cTerminus of mVenus (aa 155-239) | pGGD000 | oJM110/oJM111 | pRD43 |
| pJM134/cDNA_SEP3 | *SEPALLATA3* cDNA full length | pGGC000 | oJM116/oJM117 | L-*er* cDNA |

**Tab. S6. Plant expression plasmids constructed in this study.** Plasmids were used for stable *A. thaliana* transformation or transient transformation of *N. benthamiana*. Construction of the plasmids was achieved by the GreenGate method using the appropriate Inserts and assemble them in the respective Backbone.

| **ID** | **Inserts** | **Backbone** |
| --- | --- | --- |
| pJM33/UBcdsAP1mVenus | pGGA006, pGGB003, pJM9, pRD43, pGGE009, pGGF008 | pGGZ001 |
| pJM34/UBcdsAP1mCherry | pGGA006, pGGB003, pJM9, pRD53, pGGE009, pGGF008 | pGGZ001 |
| pJM35/UBcdsAP1mTurquoise | pGGA006, pGGB003, pJM9, pBLAD011,pGGE009, pGGF008 | pGGZ001 |
| pJM39/UBcdsAP3mVenus | pGGA006, pGGB003, pJM5, pRD43,pGGE009, pGGF008 | pGGZ001 |
| pJM40/UBcdsAP3mCherry | pGGA006, pGGB003, pJM5, pRD53, pGGE009, pGGF008 | pGGZ001 |
| pJM41/UBcdsAP3mTurquoise | pGGA006, pGGB003, pJM5, pBLAD011, pGGE009, pGGF008 | pGGZ001 |
| pJM46/UBcdsPImVenus | pGGA006, pGGB003, pJM12, pRD43, pGGE001, pGGF008 | pGGZ001 |
| pJM47/UBcdsPImCherry | pGGA006, pGGB003, pJM12, pRD53, pGGE001, pGGF008 | pGGZ001 |
| pJM48/UBcdsPImTurquoise2 | pGGA006, pGGB003, pJM12, pBLAD011, pGGE001, pGGF008 | pGGZ001 |
| pJM52/XVEcdsAP1mVenus | pBLAA001, pGGB003, pJM9, pRD43, pGGE001, pGGF008 | pGD283 |
| pJM53/XVEcdsAP1mCherry | pBLAA001, pGGB003, pJM9, pRD53, pGGE001,pGGF008 | pGD283 |
| pJM54/XVEcdsAP1mTurquoise | pBLAA001, pGGB003, pJM9, pBLAD011, pGGE001, pGGF008 | pGD283 |
| pJM63/gAP1mVenus | pJM8, pGGB003, pJM9, pRD43, pGGE001, pGGF003 | pGGZ001 |
| pJM64/gAP1mCherry | pJM8, pGGB003, pJM9, pRD53, pGGE001, pGGF003 | pGGZ001 |
| pJM65/gAP1mTurquoise2 | pJM8, pGGB003, pJM9, pBLAD011, pGGE001,pGGF003 | pGGZ001 |
| pJM69/gAP3mturqoise2 | pGGA002, pGGB003, pJM5, pBLAD011, pGGE009, pGGF002 | pGGZ001 |
| pJm73/gPImturqoise2 | pJM11, pGGB003, pJM12, pBLAD011, pJM1, pGGF007 | pGGZ001 |
| pJM128/UBQ10_mCherryNLS | pGGA006, pGGB002, pGGC015, pGGD007, pGGE009, pGGF003 | pGGZ001 |
| pJM152/tUBQ10::cDNASEP3-mVenus | pGGA006, pGGB002, pJM134, pRD43, pGGE009, pGGF002 | pGGZ001 |
| pJM157/AP3-mVenusSplit1 | pGGA006, pGGB002, pJM5, pJM81, pGGE009, pGGF008 | pGGZ001 |
| pJM158/PI-mVenusSplit2 | pGGA006, pGGB002, pJM12, pJM82, pGGE009, pGGF008 | pGGZ001 |

**Tab. S7. Oligonucleotides used in this study**

| **ID** | **Sequence in 5' - 3' orientation** | **Application** |
| --- | --- | --- |
| oJM5_AP3CDS_fwd_BsaI | ATATGGTCTCAGGCTCAATGGCGAGAGGGAAGATCCAGATC | Cloning |
| oJM6_AP3CDS_rev_BsaI | ATATGGTCTCACTGATTCAAGAAGATGGAAGGTAATGATGTCAGAG | Cloning |
| oJM7_AP1CDS_fwd_BsaI | ATATGGTCTCAGGCTCAATGGGAAGGGGTAGGGTTCAATTG | Cloning |
| oJM8_AP1CDS_rev_BsaI | ATATGGTCTCACTGATGCGGCGAAGCAGCCAAG | Cloning |
| oJM9_AP1pro_fwd_BsaI | ATATGGTCTCAACCTCAATATAATGTTTAACATCCAAGATTTGTTTTACATAA | Cloning |
| oJM10_AP1pro_rev_BsaI | ATATGGTCTCATGTTTTTTGATCCTTTTTTAAGAAACTTCTTACTCTAAAAG | Cloning |
| oJM11_PICDS_fwd_BsaI | ATATGGTCTCAGGCTCAATGGGTAGAGGAAAGATCGAGATAAAGAG | Cloning |
| oJM12_PICDS_rev_BsaI | ATATGGTCTCACTGAATCGATGACCAAAGACATAATCTTTTCCTG | Cloning |
| oJM13_PIpro_fwd_BsaI | ATATGGTCTCAACCTTCACACTCGAAACCTAGTTATGTGTTTG | Cloning |
| oJM14_PIpro_rev_BsaI | ATATGGTCTCATGTTCTTTCTCTCTCTATCTCTTTCTCAATTTTAGGG | Cloning |
| oJM35_pAP1_SDM_fwd | ACCCAACTTGCTCTTTGATAGTGACCTAACTAAGACAATTTTG | Site-directed mutagenesis |
| oJM36_pAP1_SDM_rev | CAAAATTGTCTTAGTTAGGTCACTATCAAAGAGCAAGTTGGGT | Site-directed mutagenesis |
| oJM37_cdsAP3_SDM_fwd | GTTCAAATCTCTTGGGAATCAGATCGAAACCACCAAGAAAAA | Site-directed mutagenesis |
| oJM38_cdsAP3_SDM_rev | TTTTTCTTGGTGGTTTCGATCTGATTCCCAAGAGATTTGAAC | Site-directed mutagenesis |
| oJM39_cdsPi_SDM_fwd | CCTCGACAAAGTCCGTGACCACCAGGTATGT | Site-directed mutagenesis |
| oJM40_cdsPi_SDM_rev | ACATACCTGGTGGTCACGGACTTTGTCGAGG | Site-directed mutagenesis |
| oJM41_pPI_SDM_fwd | GAAGAAAACATAGTACGGAAGTGACCAGAGGTTAATTAAACGA | Site-directed mutagenesis |
| oJM42_pPI_SDM_rev | TCGTTTAATTAACCTCTGGTCACTTCCGTACTATGTTTTCTTC | Site-directed mutagenesis |
| oJM47/tOCS-fwd | ATATGGTCTCACTGCATGCATCCTGCTTTAATGAGATATGC | Cloning |
| oJM48/tOCS-rev | ATATGGTCTCATAGTATTTAGGTGACACTATAGAATATCACTAGTAAGC | Cloning |
| oJM53/GgEntry_seq_fwd | GAGTTAGCTCACTCATTAGGCAC | Sequencing |
| oJM64/pcrBlunt_seq_fwd | CAACTGTTGGGAAGGGCGATC | Sequencing |
| oJM65/cdsAP1_seq1 | GGTGTATGTTTAATCTTTGAACTAGG | Sequencing |
| oJM66/cdsAP1_seq2 | GAAACCGCAGTTAGAACTCG | Sequencing |
| oJM67/cdsAP1_seq3 | CGTTTACAAGTGTTATTATAATGTGAAC | Sequencing |
| oJM68/cdsAp1_seq4 | GAGTGCAACTGTGAAAATAAC | Sequencing |
| oJM69/cdsAP1_seq5 | GATTTTCCTTTGTTGTACGGTG | Sequencing |
| oJM70/pAP1_seq1 | CTGCTTGGAAATTTAATTATTCACTAAAG | Sequencing |
| oJM71/pAP1_seq2 | CTTTTGCTCATGATCTCCATATAC | Sequencing |
| oJM72/pAP1_seq3 | GGGGTTTTTTCACACTTGAAGATC | Sequencing |
| oJM73/pAP1_sec4 | CAATGTTACTTACCCATTTCTCTTC | Sequencing |
| oJM75/GgEntry_seq_rev | CCATATGCGGTGTGAAATACC | Sequencing |
| oJM76/GgZ001_seq_fwd | GGATATATTGTGGTGTAACGTTC | Sequencing |
| oJM77/pAP3_seq1 | GTAATCATTTGGGGAAACATATAAAAG | Sequencing |
| oJM78/pAP3_seq2 | CCTCTCCACCAAATCTCTTC | Sequencing |
| oJM79/cdsAP3_seq1 | CGCAAGGTTCTCTTCATACTTTTC | Sequencing |
| oJM80/cdsAP3_seq2 | GTGATTCTCTTATCGAAATATCTCC | Sequencing |
| oJM81/cdsmTurq2_seq1 | CACTACCAGCAGAACACCCC | Sequencing |
| oJM82/pPI_seq1 | GAATTCACGACTTTTTTTGTGG | Sequencing |
| oJM83/pPI_seq2 | GCAAAATCATTGGGACACTATC | Sequencing |
| oJM84/pPI_seq3 | CATTTCTCTCTCTATCTCATCAATG | Sequencing |
| oJM85/cdsPI_seq1 | CTTGTACTCAACAAGACATGG | Sequencing |
| oJM86/cdsPI_seq2 | CCAGAACCTTAGCAATGAGATTG | Sequencing |
| oJM87/cdsPI_seq3 | CAGGAAAAGATTATGTCTTTGGTC | Sequencing |
| oJM97/cdsAP3_seq1_rev | GAAAAGTATGAAGAGAACCTTGCG | Sequencing |
| oJM108/mVenusSplit1_D_fwd | ATATGGTCTCATCAGCAATGGTGAGC | Cloning |
| oJM109/mVenusSplit1_D_rev | ATATGGTCTCAGCAGTTAGGTGATATAGACGTTGTGGCTGTTG | Cloning |
| oJM110/mVenusSplit2_D_fwd | ATATGGTCTCATCAGCAGCCGACAAGCAGAAGAACGG | Cloning |
| oJM111/mVenusSplit2_D_rev | ATATGGTCTCAGCAGTTACTTGTACAGC | Cloning |
| oJM112/mVenusSplit1_C_fwd | ATATGGTCTCAGGCTCAATGGTGAGCAAGGGCGAGG | Cloning |
| oJM113/mVenusSplit1_C_rev | ATATGGTCTCACTGAGGTGATATAGACGTTGTGGCTGTTG | Cloning |
| oJM114/mVenusSplit2_C_fwd | ATATGGTCTCAGGCTCAGCCGACAAGCAGAAGAACGG | Cloning |
| oJM115/mVenusSplit2_C_rev | ATATGGTCTCACTGATTACTTGTACAGCTCGTCCATGC | Cloning |
| oJM116/cdsSEP3_fwd | ATATGGTCTCAGGCTCAATGGGAAGAGGGAGAGTAGAATTGAAG | Cloning |
| oJM117/cdsSEP3_rev | ATATGGTCTCACTGAAATAGAGTTGGTGTCATAAGGTAACCAAC | Cloning |
| oJM115/mVenusSplit2_C_rev | ATATGGTCTCAACCTCTCATGATCTTGATCCCCTGCGC | Cloning |
| M13_F | TGTAAAACGACGGCCAG | Sequencing |
| M13_R | CAGGAAACAGCTATGAC | Sequencing |
| oJM178/pistillata-1_genotyping_fwd | gagagagaaagatgggtagaggaaag | Genotyping |
| oJM179/pistillata-1_genotyping_rev | gcatctagggttaaagattcaaggg | Genotyping |

## Materials and Methods

### Cloning

#### Generation of Entry plasmids

The “entry” plasmids were cloned to assemble the expression plasmids via the GreenGate method (Lampropoulos et al. 2013). For each “entry” plasmid, the respective insert was amplified with the according primer pair from the appropriate template DNA (Sup. Tab. 5). For pJM1, pJM2, pJM5, pJM8, pJM9, pJM11, pJM12, pJM81, pJM82 and pJM134, the inserts were cloned by restriction and ligation with *Eco31*I (Thermo Scientific). *Eco31*I restriction sites contained in the promoter of APETALA1 (oJM35_pAP1_SDM_fwd; oJM36_pAP1_SDM_rev) and PISTILLATA (oJM41_pPI_SDM_fwd; oJM42_pPI_SDM_rev) and in the genomic region of APETALA3 (oJM37_cdsAP3_SDM_fwd; oJM38_cdsAP3_SDM_rev) and PISTILLATA (oJM40_cdsPi_SDM_rev; oJM39_cdsPi_SDM_fwd) were removed by site directed mutagenesis using the QuikChange II Site-Directed Mutagenesis Kit. Oligos used for amplification of promoters, coding sequences and genomic regions can be found in Sup. Tab. 7.

#### Generation of plant transformation plasmids

The plant expression plasmids used in this thesis for stable transformation of *A. thaliana* and transient expression of *N. benthamiana* were assembled via the GreenGate method which is based on Golden Gate method (Lampropoulos et al. 2013). “Entry” plasmids (Plasmids can be found in

Tab**. S2. Summary of the FRET-FLIM measurements for the investigation of MADS-domain protein interactions in *Arabidopsis*.** Mean, standard deviation (SD) and standard error (SE) of FRET efficiencies were calculated after removing values with BINDING < 10%.

| **Sample** | **mean BINDING [%]** | **SD BINDING [%]** | **SE BINDING [%]** | **mean FRET efficiency [%]** | **SD FRET efficiency [%]** | **SE FRET efficiency [%]** |
| --- | --- | --- | --- | --- | --- | --- |
| AP1-mV | -0.54 | 3.91 | 0.66 | NA | NA | NA |
| AP1-mV AP1-mCh | 8.59 | 5.37 | 0.98 | 56.67 | 19.52 | 5.04 |
| AP1-mV PI-mCh | 3.28%± | 3.91 | 1.04 | NA | NA | NA |
| AP3-GFP | 2.08 | 4.79 | 1.05 | NA | NA | NA |
| AP3-GFP AP1-mCh | 4.3 | 2.47 | 0.78 | NA | NA | NA |
| AP3-GFP PI-mCh | 3.83 | 2.41 | 0.53 | NA | NA | NA |
| PI-GFP | 2.36 | 4.7 | 0.94 | NA | NA | NA |
| PI-GFP AP1-mCh | 4.99 | 2.95 | 0.59 | 64.91 | NA | NA |
| SEP3-GFP | -1.88 | 5.67 | 1.13 | 11.24 | NA | NA |
| SEP3-GFP AP1-mCh | 0.67 | 3.68 | 0.82 | NA | NA | NA |

Sup. Tab. 3, Sup. Tab. 4 and Sup. Tab. 5) were used to combine multiple elements by repeated restriction and ligation. In Sup. Tab. 6 all assembled expression plasmids are listed. Each plasmid was created by running a Greengate reaction as described by Lampropoulos et al. (Lampropoulos et al. 2013) with the listed Inserts and the respective plasmid backbone. For all GreenGate reactions, the number of cycles was increased to 50. Eco31I (Thermo Scientific) was used for restriction and a T4-Ligase (Thermo Scientific) was used for ligation.

### Generation of stable transgenic *A. thaliana* plants

A ~3kb promoter region upstream of the AP1 start codon was used to drive expression of AP1-mT2, AP1-mV or AP1-mCh fusion proteins and a ~2kb promoter region upstream of the PI start codon was used to drive expression of the PI-mCh reporter.

Plants were transformed with the floral dip method as described by Zhang and colleagues (Zhang et al. 2006). Briefly, a 500 ml main culture of transformed *Agrobacterium tumefaciens* was inoculated Material and Methods 35 from a 5 ml preculture and grown for 1 day at 28°C. Bacteria were harvested at 4000 x *g* for 10 min and resuspended in 500 ml dipping solution (0.02 % (v/v) Silwet; 5 % (w/v) sucrose). Inverted plants were dipped for 2-5 min into the bacteria containing solution. To maintain high humidity, dipped plants were covered with foil or kept in a closed plastic bag in the dark for 16-24 h, before they were transferred back to light conditions. AP1 reporter constructs were transformed in *ap1-1* mutant plants. The PI-mCh reporter construct was transformed in in L-*er* plants and crossed into the *pi-1* mutant background for complementation assay. All FP fusion constructs were able to rescue the respective stamen and/or petal deficiency of the *pi-1* or *ap1-1* mutant (Sup. Fig. 9). Plants of the L-*er* ecotype were dipped in a desiccator, where a vacuum was applied. Double or triple marked reporter lines were generated by successive crossing of the individual single reporter lines. The p*PI*::*PI*-GFP, p*AP3*::*AP3*-GFP and p*SEP3*::*SEP3*-GFP reporters were previously described (Wuest et al. 2012) (de Folter et al. 2007).

### Plant selection

For plant selection on soil, Basta™ (glufosinate-ammonium; Bayer CropScience Deutschland GmbH, Langenfeld, Germany) was sprayed on one-week old seedlings. The Basta™ concentration was 20 ml/L. Selection with D-Alanine, hygromycin and kanamycin was performed on GM plates. Respective concentrations were 4 mM, 50 µg/ml and 25 µg/ml.

### Growth conditions

For seed amplification, Basta™ selection and microscopy, *A. thaliana* plants were grown at 21°C on soil in a Phyto chamber with long day conditions (16 h light). For plant selection with kanamycin, hygromycin and D-Alanine, pants were grown under continuous light at 21°C on GM-plates containing the respective chemical. After two- or three-weeks positive plants were transferred to soil and grown at 21°C in a Phyto chamber with long day conditions (16 h light). *N. benthamiana* plants used for transient expression were grown in the greenhouse for four weeks prior to transformation

### Transient transformation of *N. benthamiana*

For transient transformation of N. benthamiana, an overnight culture of agrobacteria was inoculated in DYT and incubated on 28°C one day before infiltration. Bacteria were then centrifuged at 4000 x g and resuspended in infiltration medium (5 % (w/v) saccharose; 0.01 % (w/v) MgSO_4_; 0.01 % (w/v) glucose; acetosyringone 450 µM) to an O.D._600_ of 0.4-0.8. Resuspended bacteria were kept on ice for 1-2 h prior to infiltration. For co-infiltration, bacteria solutions were mixed in equal quantities and Agrobacterium strain GV3101:p19 expressing the p19 silencing inhibitor was added to each mix. Infiltration of the bacteria solution was performed with a 1 ml syringe on to the abaxial side of 3‐ to 4‐week‐old plants. Plants were kept under continuous light before microscopy analysis. For ß-estradiol-inducible constructs, plants were sprayed on the abaxial side of infiltrated leaves with an estradiol solution (ß-estradiol 20 µM; Tween-20 0.1 %) prior to imaging.

### Fluorescence imaging

For fluorescence imaging a Zeiss LSM 780 confocal microscope (40× water immersion objective, Zeiss C‐PlanApo, NA 1.2) was used. mVenus was excited at 514 nm; GFP at 488 nm; mCherry at 561; mTurquoise2 at 440. Signal recording of each fluorophore was done within its maximum emission peak while avoiding auto-fluorescence above 650 nm. The fluorescent properties of the fluorophores used here are part of a “protein collection” (Denay et al. 2019) described at <https://www.fpbase.org/collection/332/>.

Cell walls were stained with either propidium iodide (PI 5 mM) or 4′,6-Diamidin-2-phenylindol (DAPI 1 µg/ml). PI was excited at 561 nm and recorded at 595 - 650 nm; DAPI was excited at 405 nm and recorded at 410 - 435 nm.

For photobleaching-FLIM, images of nuclei of co-filtrated plants were acquired before and after photobleaching of the acceptor. Photobleaching of mCherry was performed with a 561 nm laser at 100% for ninety frames.

### Fluorescence Lifetime imaging

Fluorescence lifetime was measured at a Zeiss LSM 780 confocal microscope (40× water immersion objective, Zeiss C-PlanApo, NA 1.2). For TCSPC a PicoQuant HydraHarp 400 (PicoQuant, Berlin, Germany) was used. Photon counting was performed with a picosecond resolution. mVenus and GFP were excited with a 485 nm (LDH‐D‐C‐485, 32 MHz, PicoQuant, Berlin, Germany) pulsed polarized laser. Laser power at the objective lens was adjusted to 1 µW in *N. benthamiana* experiments or 1.4 µW in Arabidopsis experiments. Light, emitted from the sample, was separated by a polarizing beam splitter before photons were selected with a band-pass filter. For mTurquoise2 a 482/35 band-pass filter was used, for GFP a 520/30 band-pass filter, for mVenus a 534/30 band-pass filter, and for mCherry a 607/70 band-pass filter. When GFP and mVenus served as a donor, a LP610 beam splitter was used. Photons were detected in both donor and acceptor channel simultaneously with Tau‐SPADs (PicoQuant, Berlin, Germany). Images were acquired at zoom 8, zoom 4 or zoom2 with a resolution of 256x256pixel (*N. benthamiana* and *Arabidopsis*) or 512x512 pixel (*Arabidopsis*) with a pixel size of 0.1 µm or 0.41 µm, respectively, and a pixel dwell time of 12.54 µs and laser repetition rate of 32 MHz. Photons were collected over 60 frames in experiments with *N. benthamiana*, and over 80 frames during experiments with *A. thaliana*. To avoid pileup effects, nuclei containing high donor concentrations were avoided. Before image acquisition, the system was calibrated for each donor. For this, the objective was adjusted to reach a maximal count rate. FCS curves of Rhodamine110 dye (for GFP or mVenus donors) and water were acquired to monitor the system function. Internal response functions for each laser were determined by measuring the fluorescence decay of quenched erythrosine in saturated KI for GFP or mVenus donors, using the same hardware settings as for the FRET pair of interest.

### Fitting of the fluorescence decays

#### One pattern analysis for bi-exponential decaying donor

The fluorescence decays of selected ROIs in the FLIM image were analysed with the SymPhoTime FLIM analysis software (SymPhoTime 64, version 2.4; PicoQuant, Berlin, Germany). TCSPC bins of channel 1 and 2 (parallel and perpendicular light) were binned by sixteen resulting in a bin width of 16 ps. Nuclei were selected by hand using the ROI tool. Chloroplasts and pixels above the pile-up limit (10 % of the laser repetition rate, >2,421 counts per pixel accumulated over 60 frames with a pixel dwell time of 12.54 µs) were manually removed. Decays from donor only samples were fitted with the FLIM analysis tool (Fitting model: n-exponential reconvolution). Judged by fitting residuals and Chi-square-test, two lifetimes (model parameter n=2) were needed to fit donor only decays containing either mVenus or GFP. mVenus decays were consisting of a longer lifetime of ~3 ns and a shorter lifetime of ~1-2 ns. GFP decays were consisting of a longer lifetime of ~2.6-2.8 ns and a shorter lifetime of ~0.1-1 ns.

FRET samples were fitted using the FLIM One Pattern analysis tool (Fitting model: n-exponential reconvolution) with 3 model parameters.

This tool is a special script, which can be implemented into SymPhoTime 64 and can be obtained by PicoQuant on request. As a standard, SymPhoTime 64 includes a tool to analyse FRET efficiency and BINDING for monoexponential decaying FRET donors. Since many FRET donors exhibit bi-exponential decays, the “One pattern” analysis tool was developed. In a first experiment, donor only decays are fitted as described above and their two lifetime components as well as the ratio of the amplitudes of both components are determined. In a second step, the FRET decay is fitted with a model comprising of three lifetime components. The donor lifetime 1 and 2 are entered into the model as measured beforehand in the donor only samples. The amplitude of lifetime 1 is set to 1.0 while the amplitude of lifetime 2 is set to the amplitude fraction determined in the donor only experiment. These two fixed lifetimes and their amplitude ratio form a so called “pattern”. During the fit, only the height of the pattern is adapted. This is performed by a proportionality parameter. It is multiplied with the pattern and is adapted during the fit to reach the best agreement between fit and experimental data.

Together with this proportionality parameter, a third lifetime and its amplitude are fitted to describe the lifetime decay of the FRET species.

Although there is the possibility that the FRET species decay would also be bi-exponential, as the Donor only is described by a bi-exponential decay. However, in all cases measured we found good agreement between fit and experimental data by using only a mono-exponential decay for the FRET species.

The third lifetime is corresponding to the FRET fraction of the sample and was fitted within limits corresponding to 10% and 80% of the longer lifetime acquired in the donor samples and was used to calculate FRET efficiencies as the lifetime of the FRET sample over the arithmetic mean of the longer lifetimes of the respective donor‐only samples:

$${FRET}_{eff}=1-\left( \frac{{}_{FRET}}{\overline{{}_{donor}}} \right)$$

The average of the longer lifetime component measured in several donor-only samples was used as donor lifetime in the FRET efficiency equation.

The relative amplitude of the FRET fraction (amplitude of the FRET fraction divided by the sum of all amplitudes of the fit) corresponds to the size of the FRET fraction and displays the percentage amount of donor molecules bound to acceptor molecules in the sample (termed BINDING [%]). Here, we considered BINDING as a measure for the affinity of the interaction and the FRET efficiency as a measure of the strength of the interaction between the fluorophores in the complex. Decays of donor only samples and NLS-mCherry negative control samples were also fitted using the fitting routine for FRET samples to define a BINDING limit (marked by a blue dashed line in the resulting summarizing box plots). We realized that below this limit the FRET efficiencies calculated based of the fits are close to the selected limits (10% and 80%) and the distribution of the FRET efficiency results is very broad, as the low photon statistics for the FRET component is no not sufficient to stably fit a lifetime. Therefore, we excluded FRET efficiencies that show BINDING values below 10% in the box plots.

The determination of the BINDING parameter, which is derived from the amplitude, is much more stable than the determination of the lifetime parameter and can be obtained even at low photon numbers of the FRET population with acceptable accuracy.

Fitting formula for the FRET species:

$$y\left( t \right)=P_{12}\left( a_{1}exp\left( \frac{-t}{\tau_{1}} \right)+a_{2}exp\left( \frac{-t}{\tau_{2}} \right) \right)+a_{3}exp\left( \frac{-t}{\tau_{3}} \right)+Bkgr$$

P_12_ is the proportionality fitting parameter for the fixed biexponential decay of the donor only. It adapts the amplitude of the fixed biexponential donor decay to the measured TCSPC curve of the FRET sample.

The amplitude of the first component of the biexponential decay of the donor a_1_ is set to 1. A_2_ is set to the ratio a_2_^m^/ A_1_^m^ of the measured amplitudes a_i_^m^, I = 1, 2 of the biexponential decay of the donor. τ_1_ is set to the longer lifetime of the biexponential decay of the donor, while τ_2_ is the shorter one. In this way, A_2_,τ_1_, τ_2_ are not fitted but determined in a separate measurement of the donor only sample.

For monoexponential fitting of the FRET fraction n is set to n = 3. In this way A_3_ and τ_3_  are the fitting parameters for the FRET fraction. Background (Bkgr) is fitted to take account for the residual background of the decay curve at long times t.

The relative amplitude of the FRET fraction a_rel3_ is determined by the following formula:

$$BINDING=a_{rel3}=\frac{a_{3}}{P_{12}\left( a_{1}+a_{2} \right)+a_{3}}$$

All parameters are set / determined as stated above.

Parts of this method is covered by a German patent application DE10 2021 107 759.1.

#### Lifetime FRET analysis for mono exponential decaying donors

The fluorescence decays of selected ROIs in the FLIM image were analysed with the SymPhoTime FLIM analysis software (SymPhoTime 64, version 2.4; PicoQuant, Berlin, Germany). TCSPC bins of channel 1 and 2 (parallel and perpendicular light) were binned by sixteen resulting in a bin width of 16 ps. Nuclei were selected by hand using the ROI tool. Chloroplasts and pixels above the pile-up limit (10 % of the laser repetition rate, >2,421 counts per pixel accumulated over 60 frames with a pixel dwell time of 12.54 µs) were manually removed. Decays from donor only samples were fitted with the FLIM analysis tool (Fitting model: n-exponential reconvolution) and only one model parameter (n=1) was used. FRET samples (containing mVenus and mCherry) as well as donor only samples (only mVenus) were fitted using the “LT FRET image” analysis tool. Parameter “${}_{F donor}$” was fixed as the average donor only lifetime measured beforehand in the FLIM analysis.

The second lifetime parameter “${}_{FRET}$” is corresponding to the FRET fraction of the sample and was fitted within limits corresponding to 10% and 80% of the average lifetime acquired in the donor only samples.

BINDING corresponds to the relative amplitude of the FRET fraction:

$$BINDING \left( rel. a_{FRET} \right)=\left( \frac{a_{FRET}}{a_{Donor}+ a_{FRET}} \right)$$

FRET efficiencies were calculated as the lifetime of the FRET sample over the arithmetic mean of lifetimes of the donor‐only samples measured beforehand:

$${FRET}_{eff}=1-\left( \frac{{}_{FRET}}{\overline{{}_{donor}}} \right)$$
